# Three-dimensional structure model and predicted ATP interaction rewiring of a deviant RNA ligase 2

**DOI:** 10.1186/s12900-015-0046-0

**Published:** 2015-10-09

**Authors:** Sandrine Moreira, Emmanuel Noutahi, Guillaume Lamoureux, Gertraud Burger

**Affiliations:** Department of Biochemistry and Robert-Cedergren Centre for Bioinformatics and Genomics, Université de Montréal, Montreal, QC Canada; Department of Biochemistry, currently Département d’informatique et de recherche opérationnelle (DIRO), Université de Montréal, Montreal, QC Canada; Department of Chemistry and Biochemistry, Centre for Research in Molecular Modeling (CERMM), Groupe d’étude des protéines membranaires (GÉPROM), Regroupement québécois de recherche sur la fonction, l’ingénierie et les applications des protéines (PROTEO), Concordia University, Montreal, QC Canada

**Keywords:** Protein structure, Molecular dynamics simulation, Protein evolution

## Abstract

**Background:**

RNA ligases 2 are scarce and scattered across the tree of life. Two members of this family are well studied: the mitochondrial RNA editing ligase from the parasitic trypanosomes (Kinetoplastea), a promising drug target, and bacteriophage T4 RNA ligase 2, a workhorse in molecular biology. Here we report the identification of a divergent RNA ligase 2 (DpRNL) from *Diplonema papillatum* (Diplonemea), a member of the kinetoplastids’ sister group.

**Methods:**

We identified DpRNL with methods based on sensitive hidden Markov Model. Then, using homology modeling and molecular dynamics simulations, we established a three dimensional structure model of DpRNL complexed with ATP and Mg2+.

**Results:**

The 3D model of *Diplonema* was compared with available crystal structures from *Trypanosoma brucei*, bacteriophage T4, and two archaeans. Interaction of DpRNL with ATP is predicted to involve double π-stacking, which has not been reported before in RNA ligases. This particular contact would shift the orientation of ATP and have considerable consequences on the interaction network of amino acids in the catalytic pocket. We postulate that certain canonical amino acids assume different functional roles in DpRNL compared to structurally homologous residues in other RNA ligases 2, a reassignment indicative of constructive neutral evolution. Finally, both structure comparison and phylogenetic analysis show that DpRNL is not specifically related to RNA ligases from trypanosomes, suggesting a unique adaptation of the latter for RNA editing, after the split of diplonemids and kinetoplastids.

**Conclusion:**

Homology modeling and molecular dynamics simulations strongly suggest that DpRNL is an RNA ligase 2. The predicted innovative reshaping of DpRNL’s catalytic pocket is worthwhile to be tested experimentally.

**Electronic supplementary material:**

The online version of this article (doi:10.1186/s12900-015-0046-0) contains supplementary material, which is available to authorized users.

## Background

RNA ligase from phage T4, the work horse of molecular biology research, is the best known member of a large protein family encompassing RNA and DNA ligation enzymes [1]. RNA ligases fall into three classes: (i) RNA ligases type 1, (ii) RNA ligases type 2, and (iii) capping enzymes. All nucleic acid ligases share a characteristic nucleotidyltransferase domain in their N-terminal part with five conserved motifs (I, III, IIIa, IV and V) [2]. Two other classes of enzymes that have RNA ligase activity but lack the above structural features are the LigT phosphoesterases involved in RNA splicing [3–5] and the recently identified RtcB proteins [6, 7]. In the following, the term “RNA ligase family” will refer to the two former classes that contain a nucleotidyltransferase domain.

RNA ligase 1 enzymes are mainly present in viruses, mammals and fungi [8]. This enzyme class is typically involved in defense as exemplified by its founding member, the phage T4 RNL1, which is deployed in the counter-attack against antiviral strategies of bacteria [9], but is also involved in tRNA intron splicing [10] and in the unconventional splicing initiating the unfolded protein response of the endoplasmatic reticulum. RNA ligases 2 have a broad but punctuated distribution across the tree of life [8]: they are found mainly in viruses -with the archetypical example of T4 RNA ligase 2 [11]- and bacteria, while only a few examples are known in archaea and eukaryotes. The biological role of RNA ligases 2 is unknown, except for the members of kinetoplastids [12].

Kinetoplastids (Euglenozoa) are a group of protozoans, some members of which are causing life-threatening human diseases (leishmaniasis, Chagas disease, sleeping sickness) [13]. These species also display a unique mitochondrial genome structure composed of an intricate network of large and small circular chromosomes [14]. Large chromosomes encode typical mitochondrial protein-coding genes. Small circles specify guide RNAs that serve as proofreading templates for editing pre-mRNAs of mitochondrial genes [15, 16]. Editing proceeds by cutting the pre-mRNA molecule at the place of the mismatch, then adding or removing uridines, and finally religating the two parts of the RNA molecule. It is this last step that is performed by RNA ligase 2. Specifically, two different RNA ligases 2 are involved, one dedicated to adding and the second to deleting uridines as exemplified by the ligases TbREL1 and TbREL2 respectively for *Trypanosoma brucei* [17].

Here we report the identification of a putative new member of the RNA ligase 2 family in *Diplonema papillatum*, a member of diplonemids (Euglenozoa), which are the sister group of kinetoplastids. The corresponding gene was discovered in our search of a candidate enzyme involved in the eccentric post-transcriptional processing in *Diplonema* mitochondria [18, 19]. This protist harbors a highly complex mitochondrial genome sharing certain similarities with that of kinetoplastids. First, the *Diplonema* mitochondrial DNA (mtDNA) is also multi-partite, as it is composed of hundreds of circular chromosomes of two size classes. The difference and uniqueness of the diplonemid mtDNA is that each chromosome contains one short coding region specifying a fragment of a gene. Each gene module is transcribed separately and then trans-spliced to form full-length mRNAs or structural RNAs. The second resemblance with kinetoplastid mitochondria is RNA editing [18, 20]. Uridine insertion and deletion editing in kinetoplastids involves an RNA ligase 2 to reseal the transcript. In *Diplonema*, RNA editing proceeds by uridine appendage at certain module ends, prior to trans-splicing. We hypothesize that an ancestral molecular machinery containing RNA ligase 2 has led to the editosome in kinetoplastids, while it has evolved to perform trans-splicing in the diplonemid branch.

RNA ligases 2 consist of two discrete portions: the N-terminal nucleotidyltransferase domain (amino acids 1–234 in T4) and a C-terminal domain (amino acids 244–329 in T4) responsible for substrate specificity. The ligation reaction of RNA ligase 2 is ATP and Mg^2+^ dependent [10, 21, 22] and proceeds, like all members of the DNA/RNA ligase family, in three steps. During the first step, ATP adenylates the enzyme on the lysine residue of the conserved KxxG tetramer in motif I of the nucleotidyltransferase domain. In step 2, the covalently linked AMP is transferred to the 5′P of the ‘downstream’ RNA molecule to be ligated. Finally, the 3′OH of the ‘upstream’ RNA molecule attacks the 5′P of the ‘downstream’ RNA by releasing AMP and joining the two RNA molecules (Additional file 1: Figure S1). The crystal structure has been determined for only a few family members, notably T4 RNA ligase 2 [23, 24] and one of the two paralogous mitochondrial RNA ligases 2 from *Trypanosoma brucei*, notably in apo form as well as complexed with a magnesium ion and ATP [25].

In this study, we devise a strategy based on hidden Markov models (HMMs) and structural comparisons to identify proteins of large evolutionary distance to well-studied counterparts in model organisms. Comparative analysis of highly diverged homologs is particularly informative for identifying functionally and structurally important residues that are under elevated selective pressure. Employing this analytic strategy, we identify the gene and model the structure and ligand interactions of a putative RNA ligase 2 from *Diplonema*. The model predicts intriguing innovations in the interaction network between ATP and the residues of the catalytic pocket, which are worthwhile to be tested experimentally by resolving the crystal structure. We discuss possible evolutionary scenarios that led to these innovations.

## Results

### HMM-based detection of a divergent RNA ligase 2 in *Diplonema*

In general, proteins of *D. papillatum* display a low level of sequence similarity with homologs of other taxa, and are difficult to identify with tools based on sequence similarity such as BLAST [26]. Therefore we employed more sensitive methods based on Hidden Markov Models (HMMs). We used the HMM PF09414.4 from the Protein FAMily database (PFAM) [27], a model that was built based on RNA ligases 2 from all domains of Life including mitochondrial RNA ligases 2 of kinetoplastids. We identified one candidate protein, Dp28902_3, in the conceptual translation of the *Diplonema* draft genome assembly (version no. 2). Expression of this open reading frame was confirmed by RNAseq experiments. The corresponding transcript is poly-adenylated and its steady-state level is about 1/10 compared to the expression of Aspartyl tRNA synthase.

For comparison, we also used HMMs for other RNA and DNA ligase super-families in searches against Dp28902_3 and RNA ligases 2 of *Trypanosoma* (TbREL1, positive control) and the heterolobosean *Naegleria gruberi*. *Naegleria* was chosen because heteroloboseans are the sistergroup of Euglenozoa, and because sequences of this taxon have not been used in building the PFAM HMM. Table 1 summarizes the corresponding *E*-values. Dp28902_3 has the lowest *E*-value with the PF09414 model, a value that is 10^7^ times smaller than the second-best match, which was obtained with the HMM of ATP-dependent DNA ligases. Models for proteins that have a different fold (PF02834-LigT, PF01139-RtcB) did not yield significant *E*-values (>0.05) for either Dp28902_3 or the RNA ligases 2 of *Trypanosoma*. Therefore, Dp28902_3 most likely belongs to the RNA ligase 2 family and will be referred to as DpRNL.

**Table 1:** Identification of the ligase family to which belongs DpRNL^a^

Family	PFAM	*D. papillatum* DpRNL	*N. gruberi* XP_002674912.1	*T. brucei* KREL1	*T. brucei* KREL2
DNA ligase					
[N] ATP dependent	PF01068	3.30 × 10^−5^	2.60 × 10^−5^	1.00 × 10^−3^	1.60 × 10^−6^
[N] NAD dependent	PF01653	2.20 × 10^−2^	2.90 × 10^−2^	–	4.70 × 10^−1^
RNA ligase					
[N] Rnl1 defense, splicing	PF09511	2.70 × 10^−1^	1.30 × 10^−2^	3.40 × 10^−1^	4.00 × 10^−1^
**[N] Rnl2 editing**	**PF09414**	**4.90** × 10^−12^	**3.20** × 10^−9^	**7.90** × 10^−55^	**4.30** × 10^−53^
[N] Capping	PF01331	2.70 × 10^−1^	1.70 × 10^−1^	9.10 × 10^−3^	2.10 × 10^−1^
LigT	PF02834	–	–	–	–
RtcB splicing	PF01139	–	–	4.80 × 10^−1^	–

^a^Family names preceded by an [N] are those containing a Nucleotidyltransferase domain. Each model was searched with HMMer against all the proteins of *Diplonema* papillatum, *Naegleria gruberi* and *Trypanosoma brucei* TREU927. This table presents the *E*-value for the RNA ligases 2 proteins only. The line for the PFAM domain specific for RNA ligases 2 is in bold

### DpRNL contains a nucleotidyltransferase domain typical for RNA ligases 2

**Fig. 1:**
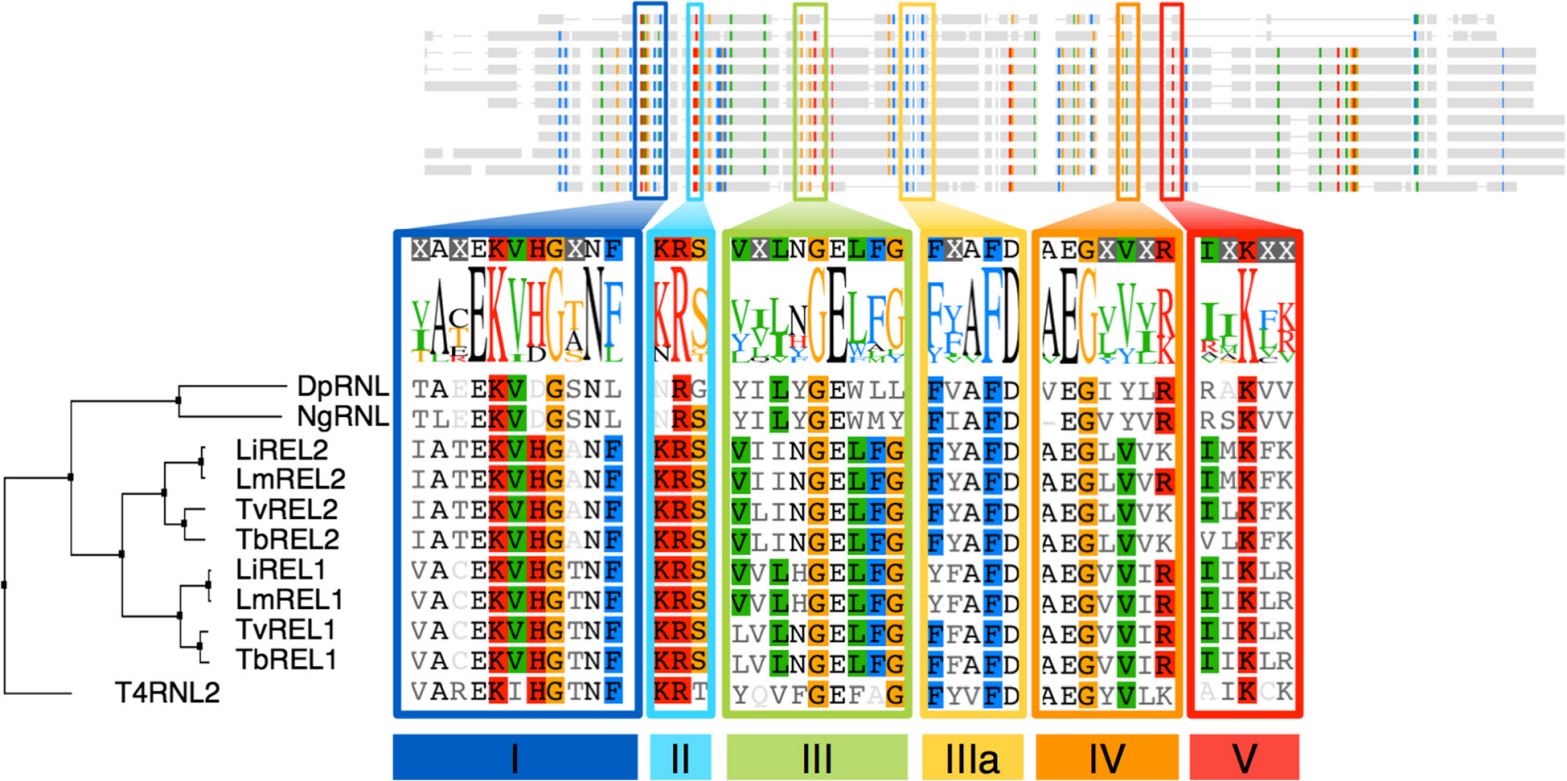
Delineation of the Nucleotidyltransferase domain. Multiple alignment of RNA ligases 2 from *Enterobacteriophage* T4 (T4RNL2), *Diplonema papillatum* (DpRNL), *Naegleria gruberi* (NgRNL), and four kinetoplastids, *Leishmania infantum* JPCM5 (LiREL1, LiREL2), *L. major* Friedlin (LmREL1, LmREL2), *Trypanosoma vivax* Y486 (TvREL1, TvREL2), and *T. brucei* TREU927 (TbREL1, TbREL2). The six sub-domains (I, II, III, IIIA, IV and V) highlighted in orange, cyan, green, blue, yellow and red, respectively are clearly detectable in DpRNL

**Fig. 2:**
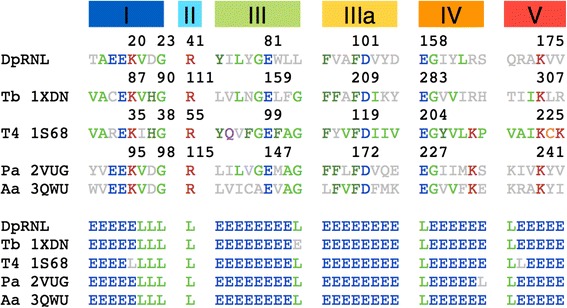
Structural alignment with DALI [59] of the *Diplonema* model (first line) and four structures: 1XDN (*Trypanosoma brucei*), 1S68 (*Enterobacteriophage T4*), 2VUG (*Pyrococcus abyssi*), and 3QWU (*Aquifex aeolicus*)

The RNA/DNA ligase super-family is characterized by a nucleotidyltransferase domain including five subdomains (motifs I, III, IIIa, IV, V) [2] located in the N-terminal portion of the protein. We demonstrate the presence of these motifs in DpRNL by three different methods: sequence alignment against PFAM HMM (Additional file 1: Figure S2); multiple sequence alignment of DpRNL and RNA ligases 2 from kinetoplastids, enterobacteriphage T4, and *Naegleria* (Fig. 1); and structural alignment of DpRNL with RNA ligases 2 for which the three-dimensional (3D) structure has been experimentally determined, notably from *Trypanosoma brucei*, the phage T4, and the archaean *Pyrococcus abyssii* (Fig. 2).

While the five subdomain motifs are well conserved across all RNA ligases 2 and readily recognizable in DpRNL, the rest of the N-terminal portion of the *Diplonema* protein shows only low sequence similarity to established RNA ligases 2 (e.g., ~18 % identity with TbREL1). DpRNL lacks portions of two loops between domains III and IIIa (TbREL1 amino acid (aa) 163–166 and aa 176–205) that are distinctive for kinetoplastid RNA ligases 2, and that have been shown to interact with RNA [25]. Also missing from DpRNL is the loop between domains IIIa and IV of TbREL1 (aa 262–282), a loop that has been predicted to interact with other proteins of the editosome [25]. Finally, the C-terminal portion of DpRNL (aa 178–203) has no recognizable resemblance with, and its length is also shorter than the corresponding region of other RNA ligases 2.

### The 3D model of apo-DpRNL possesses all structural features typical for RNA ligases 2

**Fig. 3:**
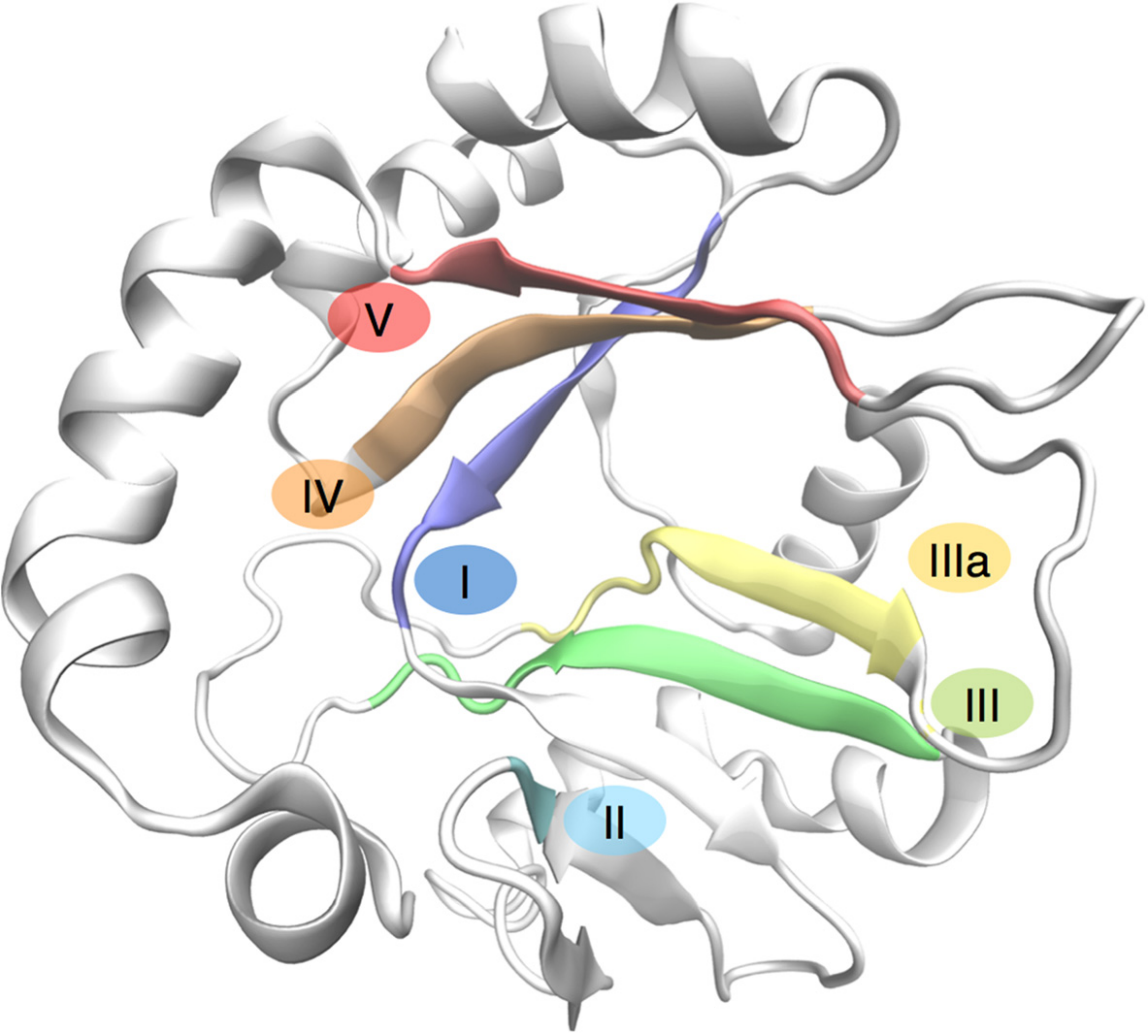
Three-dimensional model of DpRNL inferred by I-Tasser. The five Nucleotidyltransferase sub-domains are represented in color

The global three-dimensional (3D) model of DpRNL was predicted by I-Tasser [28] (Fig. 3, Additional file 2) and validated with SAVes (http://services.mbi.ucla.edu/SAVES/). Nearly all (96.1 %) amino acids have a stereochemical conformation in the “favored” or “allowed” regions of the Ramachandran plot. Only the seven most C-terminal residues are in an unfavorable environment according to the assessment by the tool Verify-3D [29]. While the per-residue analysis of ModFold [30] also found lower quality scores for the C-terminal region, the overall p-value of the model (1.547 × 10^−3^) is highly confident. The estimated TM-Score obtained from the standard output of I-Tasser was 0.70 ± 0.12. A TM-score >0.5 usually indicates a model of correct topology, and a TM-score <0.17 means a similarity no better than random. As a whole, the topology of the I-Tasser model of DpRNL is of good quality.

The 3D model of DpRNL is characterized by a core of anti-parallel-twisted β sheets decorated with apical α helices. Two structural sub-domains with similar composition are facing one another. One contains the two extremities of the molecule and consists of an anti-parallel β sheet of four β strands and four α helices. The other sub-domain, corresponding to the middle part of the protein, has six β strands and three α helices. The interface between these two sub-domains forms the catalytic pocket of the protein, with the residues of the five nucleotidyltransferase motifs pointing to the pocket’s cavity. From the inside to the outside are located motifs I, IV and V on one side, and motifs IIIa, III and II on the other, the two sides facing each other.

### Molecular dynamics simulations confirm the stability of the DpRNL 3D model

To assess the stability of the proposed DpRNL model and the relative flexibility of the structural domains, we performed a 50-ns molecular dynamics (MD) simulation. The Root Mean Square Deviation (RMSD) of the backbone α-carbon atoms remained stable after 10 ns of simulation with a mean of 4.2 Å (Additional file 1: Figure S3A).

When monitoring the secondary (2D) structure conservation during the simulation (Additional file 1: Figure S4), we observed that the β sheets, which are buried inside the protein, are more stable, whereas the α helices and loops, which are peripheral, are more flexible as reflected by the high Root Mean Square Fluctuation (RMSF) values of the corresponding residues. Specifically, certain residues of the α helices (aa 54–73 and aa 139–154) transiently adopted a 3–10 helix conformation. Flexible α helices and loops are also observed in TbREL1 of Trypanosoma, where the exposed regions of the protein interact with the RNA substrate and with other proteins of the editosome [31]. Therefore, the flexible peripheral regions of DpRNL presumably play a functional role as well.

The C-terminal region of DpRNL is linked to the rest of the molecule by a flexible loop, but this region displays less motion than expected. This is because the C-terminal domain is entangled in a network of hydrogen bonds with more N-terminal amino acids. Most stable are the interactions between the carboxyl group of tyrosine at position 203 (DpRNL_Y203, the last residue in the protein) and the lateral chain of two other residues (DpRNL_R41 with 86 % occupancy and DpRNL_S24 with 46 % occupancy), as well as between the lateral chains of DpRNL_Y203 and DpRNL_Q52. Additional stabilization of this domain comes from a hydrogen bond involving the carbonyl group of DpRNL_K202 in the main chain and the hydroxyl of DpRNL_S49. In conclusion, the 3D model of DpRNL is stable both at the 2D and 3D level. The observed flexibility parallels that of other RNA ligases 2 [24, 31], providing strong support for DpRNL being a functional member of this protein family.

### 3D structure comparison of DpRNL with well characterized RNA ligases 2

Compared to recognized RNA ligases 2, DpRNL is more conserved in 3D structure than in sequence. Nevertheless, the β strands of DpRNL are generally shorter than those of its counterparts, resulting in a 15–30 % shorter Nucleotidyltransferase domain compared to the enzymes of *Trypanosoma* or phage T4. Pairwise structural comparison with experimentally confirmed structures (Additional file 1: Table S1) reveals only a moderate fit of DpRNL with TbREL1 (RMSD of 3.4 Å), although kinetoplastids are the sister group of diplonemids. The fit is slightly better with the RNA ligases of T4 (T4RNL2; RMSD of 3.2 Å) and *Pyrococcus abyssii* (PAB1020; PDB id 2VUG; RMSD of 2.3 Å), and the putative DNA ligase from *Aquifex aeolicus* (aq_1106; PDB id 3QWU; RMSD of 2.3 Å; Additional file 3). Note that PAB1020 was initially annotated as DNA ligase, but more recent experimental studies shown that it catalyzes the ligation of RNA [32].

**Fig. 4:**
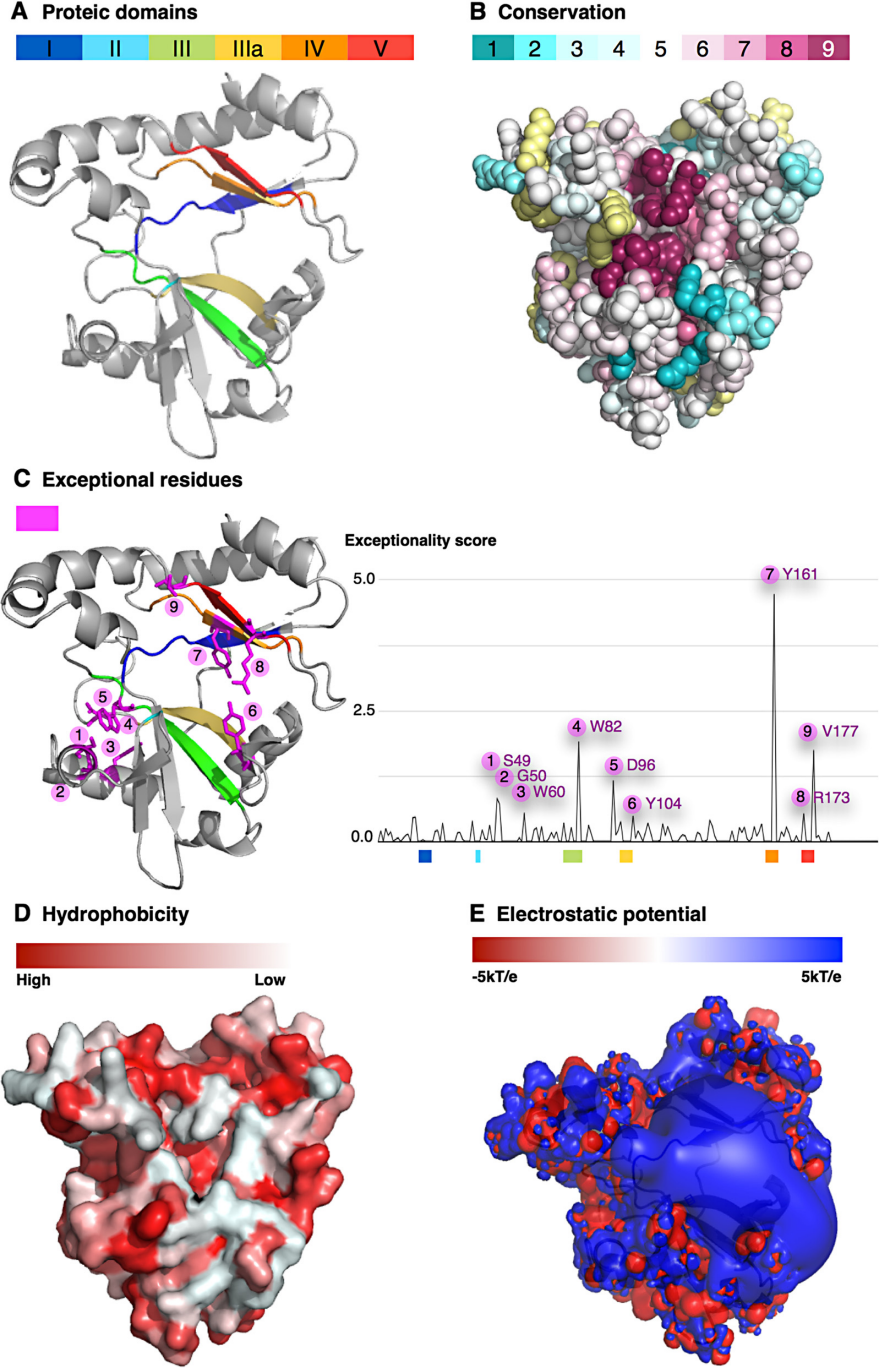
Protein properties mapped onto DpRNL. **a** Localisation of the five Nucleotidyltransferase sub-domains. **b** Amino acids conserved across the RNA ligase 2 family. The value 9 (dark purple) represents highest conservation. **c** Exceptional residues as determined in this work. **d** Hydrophobicity. **e** Electrostatic potential

The proteins from *Pyrococcus* and *Aquifex* are both homodimeric with subunits being held together through the interaction of two peripheral α helices [32]. As DpRNL has no region whose sequence resembles that of these interacting helices, we investigated if the two most C-terminal helices of DpRNL allow dimerization through typical hydrophobic interface contacts [33]. The hydrophobicity map of exposed residues (Fig. 4d and Additional file 1: Figure S5) shows that the C-terminal helices of DpRNL do not have the propensity to form an hydrophobic surface comparable to that of the archaean ligases. This suggests that DpRNL is active in a monomeric state as are TbREL1 and T4RNL2.

To determine if the Nucleotidyltransferase domain of DpRNL contains deviant residues otherwise not found in RNA and DNA ligases, we computed a score of «exceptionality» along the structural multiple alignment from selected enzymes including archaeal and kinetoplastid homologs. Each amino acid in *Diplonema* was assigned an exceptionality score based on the proportion of residues in the corresponding alignment column having common physicochemical properties in other ligases (Fig. 4c). The amino acid with the highest score is the tyrosine DpRNL_Y161, a position occupied in all other cases by a different, generally aliphatic residue. The second most deviant amino acid is the valine DpRNL_V177, whose position is generally occupied by a basic residue that non-covalently binds AMP in reaction step 1 [34]. Further exceptional residues in DpRNL are S49, G50, W60, W82, D96, Y104 and R173. The consequences of these substitutions for interactions with RNA and ATP will be discussed in a later section.

### Phylogeny of RNA ligases 2

The moderate structural similarity of DpRNL with RNA ligases 2 from the diplonemid sister group raised questions about the phylogenetic relationship of these proteins. We focused our analyses on Excavate taxa, because a broader taxonomic sampling would have resulted in sequences too diverse for meaningful phylogenetic reconstruction. The inferred tree (Additional file 1: Figure S6) shows well supported grouping of kinetoplastid RNA ligases 2, which are split into two subgroups corresponding to the two paralogs (e.g. TbREL1 and TbREL2 in *T. brucei*). The subgroup clustering strongly suggests a duplication of RNA ligases 2 in the kinetoplastid branch prior to the speciation of *Leishmania* and *Trypanosoma*. In contrast, the phylogenetic position of DpRNL in the tree has virtually no support, and the observed affiliation with a homolog from *Naegleria* (heterolobosean) might be an artifact known as long-branch attraction [35, 36]. The phylogenetic reconstruction in this instance suffers from lack of taxa within Euglenozoa (only one diplonemid, no euglenid, and no basal kinetoplastids), and from low sequence conservation. Nevertheless, the tree indicates that DpRNL diverged prior to the gene duplication event seen in kinetoplastids, and that this protein has no specific relationship to the kinetoplastid RNA ligases 2 that take part in mitochondrial RNA editing.

### DpRNL is predicted to interact with RNA in a T4-like fashion

RNA ligases 2 interact with their substrate via two regions of the protein, the C-terminal domain and regions of the N-terminal nucleotidyltransferase domain that have a positive electrostatic potential. Substrate interaction of the C-terminal domain in kinetoplastid RNA ligases 2 is indirect: the four helices bind a protein partner carrying an OB-fold that, in turn, interacts with the substrate. For example, TbREL1 recruits KREPA2, and TbREL2 associates with KREPA1 [37]. In contrast, the C-terminal domain of T4RNL2 alone suffices for efficiently binding the substrate. In DpRNL, the C-terminal domain carries only two short helices making a TbREL_KREPA-like interaction unlikely. In having a positive electrostatic potential and being rich in residues able to interact with RNA, the C-terminal domain of DpRNL resembles that of T4RNL2 [24] (Fig. 4e), and probably also interacts directly with the RNA substrate.

We mentioned earlier that the Nucleotidyltransferase domain of DpRNL lacks the two substrate-binding loops of kinetoplastid RNA ligases 2. RNA interaction of loop 1 (TbREL1 aa 167–177) and loop 2 (TbREL1 aa 190–200) had been predicted based on the crystal structure [25] and the calculation of the ensemble averaged electrostatic potential [31], and has been confirmed by an RNA ligation assay with an N-terminal fragment of TbREL1 containing these two loops [25]. The same study also shows that the equivalent N-terminal portion of T4-RNL2, which lacks these loops, does not display this activity. Again, substrate interaction in the *Diplonema* protein must be different from that in kinetoplastid RNA ligases 2 and rather similar to that of T4RNL2.

In the Nucleotidyltransferase domain of the phage T4RNA ligase 2, RNA interaction is achieved by a patch of positively charged residues located in the exposed region of central beta sheets, as revealed by the crystal structure of T4RNL2 bound to a nicked nucleic acid duplex (PDB id 2HVR). To identify such regions in DpRNL, we computed the electrostatic potential at the solvent-accessible surface of the protein (see Methods). We found a large region in DpRNL’s Nucleotidyltransferase domain with strong positive potential [23] (Fig. 4e). Superposition of the DpRNL 3D model onto the T4RNL2 structure with bound RNA duplex shows that the potential is distributed in a pattern similar to that in T4RNL2, and in addition, that the duplex broadly overlaps the positively charged regions of DpRNL (Fig. 4e). However, this region in DpRNL is not completely covered by the duplex. Either the substrate is slightly shifted and|or the unoccupied region interacts with another partner. Still, in this superposition, the two C-terminal helices of the *Diplonema* protein wrap themselves around the nucleic acid like a hook, corroborating the predicted position of the RNA substrate in the DpRNL model.

### Refinement of the DpRNL structural model by molecular dynamics simulations

RNA ligases 2 typically bind ATP in a covalent fashion during the first step of the catalysis resulting in a ligase-AMP complex (Additional file 1: Figure S1). In a previous section we reported that certain conserved residues otherwise involved in the covalent attachment of AMP, are substituted by different amino acids in DpRNL. To investigate how DpRNL might interact with ATP, we performed an MD simulation after introducing an ATP molecule together with a magnesium ion into the catalytic pocket of the 3D model to mimic the situation at the beginning of the first step of the enzymatic reaction. Our approach has been validated by a control simulation with TbREL1, where ATP and Mg^2+^ assumed stable positions in the catalytic pocket that correspond to those in the crystal structure [25].

MD simulations were performed for 50 and 45 ns. We restrained the position of ATP in the catalytic pocket during the first 15 ns (thereafter called the ATP-restrained production phase) followed by four replicates of unrestrained MD simulation during 35 ns. Second, we conducted three independent ATP-restrained productions of 15 ns, each followed by 30 ns unrestrained MD simulation in order to test whether ATP adopts each time the same position (see Additional file 1: Figure S9). We observed that the most important fluctuations during the entire simulation period took place in peripheral helices and loops, while the core β strands stabilized already during the first 10 ns (see lower RMSF values, Additional file 1: Figure S7). However, the conformation of the catalytic pocket was primarily influenced by the subtle motion of lateral chains in the core β strands that took place during the first 10-ns pre-production phase. In particular, the motion of the residues DpRNL_F101 and DpRNL_Y161, which are among the five residues with the lowest RMSF, had the strongest impact, reshaping the whole interaction network with ATP. Interestingly, DpRNL_Y161, which in the initial structure was perpendicular to ATP, turned around to face both the adenine ring and DpRNL_F101. This rotation occurred already during the MD equilibration phase, and the new position of this residue was retained for the rest of the simulation time in six of the seven replicates. A distinct conformation was adopted by the last replicate for which the number of distance violations during the ATP-restrained production phase was much higher (18 %), and ATP is more distant from both aromatic residues (5.54 Å from DpRNL_Y161 and 5.79 Å from DpRNL_F101) with a mean angle of 52° (SD = 8.8 Å) with DpRNL_F101 (Additional file 1: Figures S8, S11, Table S2). Such a conformation is incompatible with π-stacking. The conformation obtained by the six consistent simulations will be referred to as the predominant conformation and analyzed in the following sections, while the deviant conformation will be addressed in the Discussion. To summarize, in the predominant 3D model of DpRNL, the pre-production phase locked the catalytic core of the protein in a stable conformation that favors interaction with ATP.

### Predicted interactions of DpRNL with adenine and ribose of ATP

We compared the predicted interaction network of ATP in the DpRNL model with that inTbREL1, which is the only enzyme for which both the crystal structure of the protein bound to ATP (1XDN), and detailed molecular dynamics simulations are available [31]. ATP interactions of T4RNL2 are similar to those of TbREL1 (homologous residues are listed in Fig. 6) [23, 24, 31].

The phenylalanine (DpRNL_F101) and tyrosine (DpRNL_Y161), which together sequester the adenine base of ATP in the DpRNL model, establish a π-π stacking interaction with the substrate. This contrasts with the TbREL1 structure, where the base is enclosed by a sandwich composed of the aromatic ring of a phenylalanine (TbREL1_F209, motif IIIa), and a valine (TbREL1_V286). In the *Diplonema* protein the valine is replaced by a tyrosine (DpRNL_Y161), a residue determined as highly exceptional by comparative analysis (see above). This stabilizing interaction reduces greatly the degrees of freedom of the ATP molecule, and gives a significant turn to the interactions in the catalytic pocket by shifting the position of the ligand in DpRNL compared to well characterized RNA ligases. Additional ATP stabilization in DpRNL comes from two hydrogen bonds implicating the amine group of ATP. One hydrogen contacts the carbonyl group of DpRNL_E19 (equivalent to TbREL1_E86) and the other the lateral chain of DpRNL_E18 (which has no equivalent in TbREL1).

**Fig. 5:**
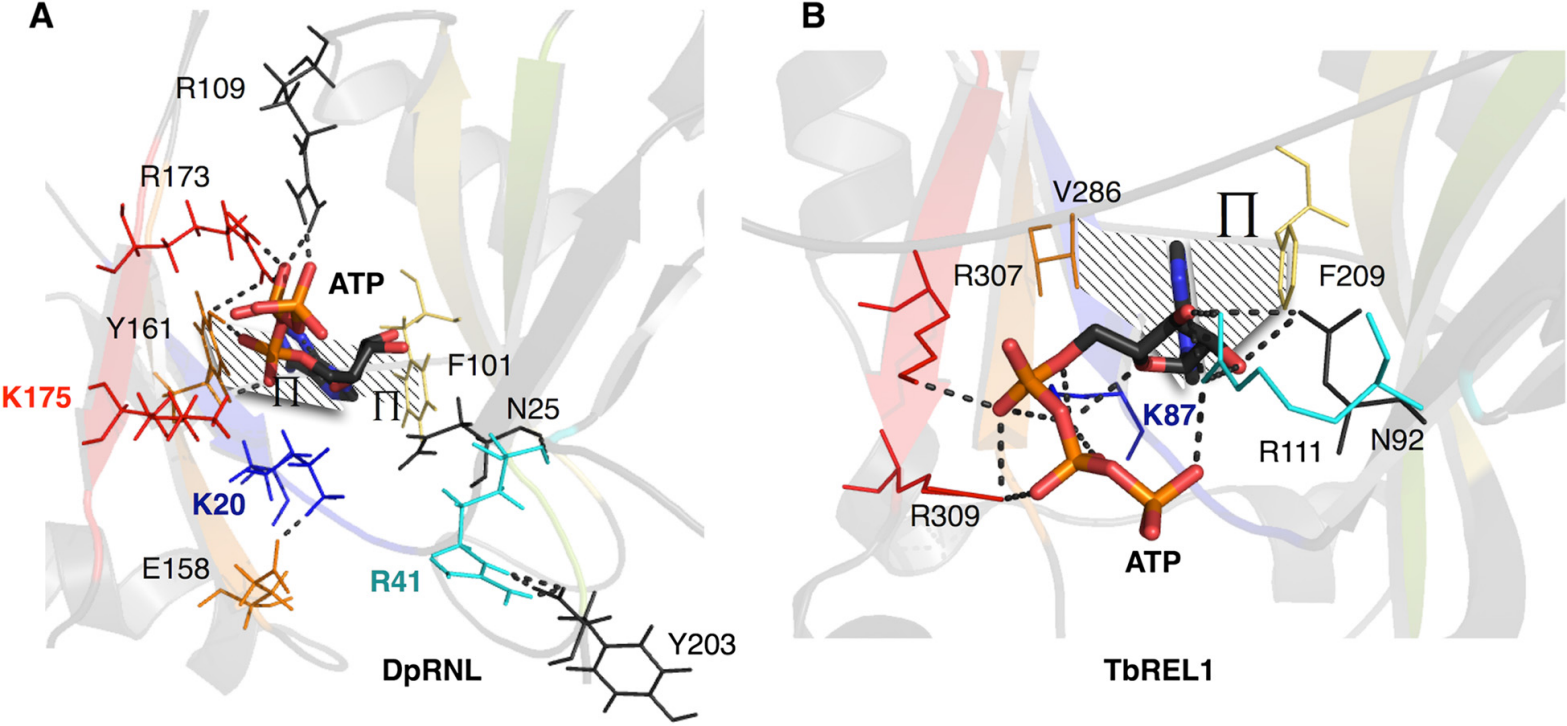
Catalytic pocket of DpRNL and TbREL1. **a** DpRNL. **b** 1XDN. Dashed lines represent interactions (π-stacking and hydrophobic) with the adenine ring. Important residues are in color

In TbREL1, the ribose of the ATP is bound by five residues (TbREL1_I59, TbREL1_K87, TbREL1_N92, TbREL1_R111, TbREL1_E159) allowing the sugar moiety only little mobility. Four out of these five residues (except TbREL1_I59) are conserved in the *Diplonema* protein (Fig. 3), but only two of the counterparts (DpRNL_N25 and DpRNL_E81) interact with the ribose of ATP (Fig. 5). Interactions in the DpRNL model take place indirectly through water molecules, and are weaker than the direct salt bridges in TbREL1, thus allowing the larger motions of the sugar that we observed. The two conserved residues that are not involved in stabilizing the sugar (DpRNL_R41 and DpRNL_K20) play an equally important role as detailed in the following.

### The triphosphate tail of ATP engages in a rich network of stabilizing interactions

**Fig. 6:**
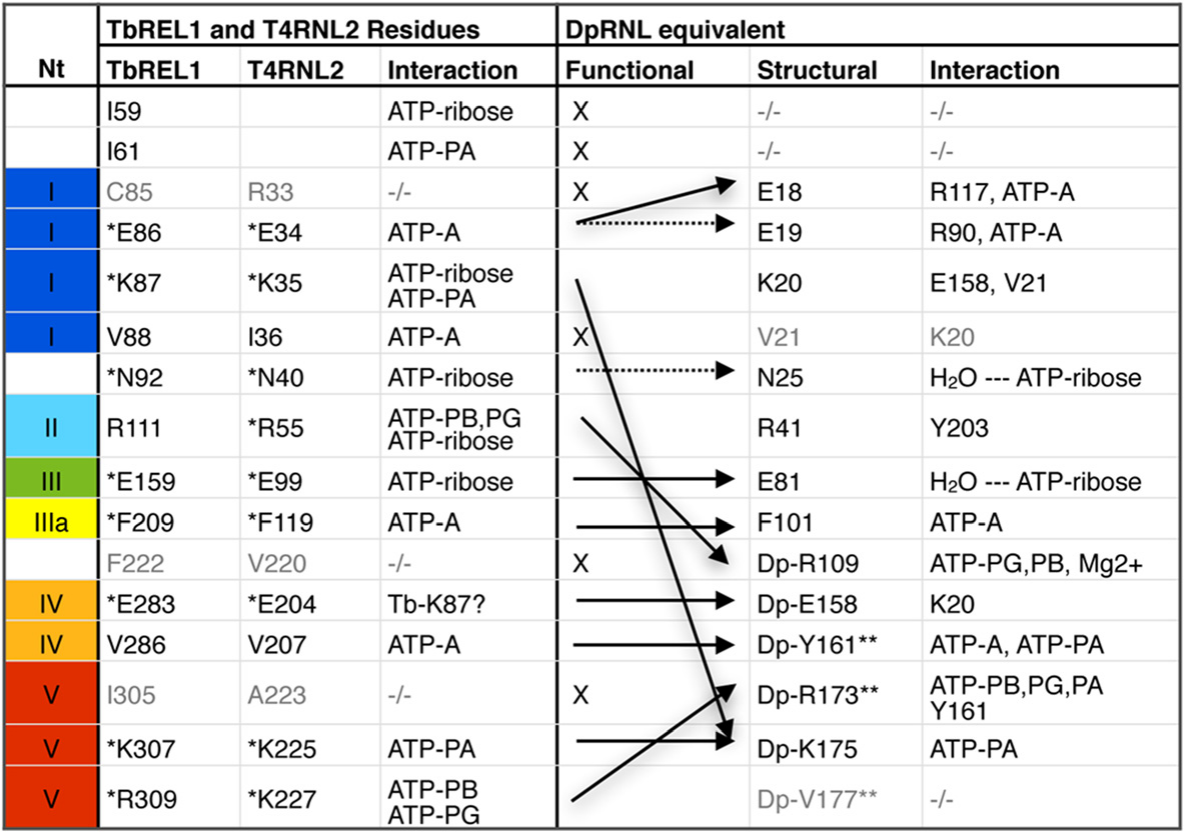
Structurally and functionally equivalent residues in DpRNL, TbREL1 and T4RNL2. Residues on the same line are structural equivalents (at the same position in a structural alignment). Residues having the same functional role are connected with an arrow. Dotted arrows indicate partial functional equivalence. X, no functional equivalent was identified. Residues in *grey* seem not to play a functional role. ATP-A: adenine of ATP; ATP-ribose: ribose of ATP; ATP-PA, PB, PG: phosphate alpha, beta, and gamma, respectively of ATP; *: essential residue; **: exceptional residue; −/−, no structural equivalent identified

In the predicted predominant conformation of DpRNL, the triphosphate tail of ATP is stabilized by a network of interactions with three basic residues (DpRNL_R109, DpRNL_R173, and DpRNL_K175). In TbREL1, the triphosphate tail is held in place by five residues, TbREL1_I61, TbREL1_K87, TbREL1_R111, TbREL1_K307 and TbREL1_R309 (see Fig. 5). Among these latter residues, only TbREL1_K307 has the same 3D position and plays the same role as predicted for DpRNL-K175, while TbREL1_I61 has no positional counterpart in DpRNL. The remaining three amino acids have a positional homolog in the DpRNL model, but apparently a different function compared to the Trypanosome protein (Fig. 6).

TbREL1_K87 is the catalytic lysine that in reaction step 1 will covalently bind ATP. This reaction is favored by strong salt bridges between ATP and several other amino acids. DpRNL_K20, the structural equivalent to TbREL1_K87, forms several salt bridges with residues DpRNL_E158, DpRNL_G159 and DpRNL_V21. But instead of promoting the covalent attachment of ATP, the interactions of DpRNL_K20 appear to rather pull this residue away from ATP, the computed distance between DpRNL_K20-Nz and Pα being on average 7.7 Å (Additional file 1: Table S2). A candidate residue for covalently binding ATP could be DpRNL_K175, owing to its position apical to the Pα at an average distance of 4.3 Å. This distance is comparable to that observed in TbREL1 between K87 and Pα. We propose that the unusual position of ATP in the DpRNL model, as well as the posited substitution of the catalytic lysine, are due to DpRNL_Y161, which, by transforming a simple to a double π-stacking interaction, shifts the position of the ligand.

TbREL1_R111 interacts with the triphosphate tail of ATP, and therefore, the functional homolog of this residue is thought to be DpRNL_R109. However, the positional counterpart of the former residue in our model (DpRNL_R41) plays a radically different role, rather forming hydrogen bridges with residues in the C-terminal region of the protein (maintained for 75.3 % of the frames). It should be stressed that all simulations with TbREL1 have been performed with a sequence lacking the C-terminal domain (because the crystal structure was determined with the N-terminal fragment of the protein), so that interactions with the C-terminal domain are not known. In T4, the crystal structure of the adenylated full-length enzyme revealed a salt bridge between two residues of the C domain, R266 and D292, probably reinforcing its structural integrity [24].

Finally, TbREL1_R309 as well interacts with the triphosphate tail of ATP, and in the homologous position of this residue, we find in the DpRNL model a valine (DpRNL_V177). However, this valine seems not to interact with ATP or any amino acid of the catalytic pocket. The functional homolog of TbREL1_R309 is rather DpRNL_R173. Note that both DpRNL_V177 and DpRNL_R173, are “exceptional” residues, and that a non-basic residue at the position corresponding to V177 in T4RNL2 was demonstrated to prevent ligation of ATP [34]). The implications of these findings will be considered in the Discussion section.

## Discussion

In the search of an enzyme responsible for the unique trans-splicing in mitochondria of diplonemids, we identified a candidate RNA ligase 2 in the *D. papillatum* genome sequence. Detection of this candidate required the most sensitive HMM search method, because molecular sequences of diplonemids are in general highly divergent [38].

To confirm the sequence-based gene assignment, we constructed a preliminary 3D model of DpRNL that we aligned with RNA ligase 2 family members. Based on the structural sequence alignment, we delineated the boundaries of the predicted functional domains of the *Diplonema* protein. To pinpoint deviant amino acids in the 3D model of DpRNL, we computed a score of exceptionality for each residue. The preliminary structural model was refined by first, eliminating structural inconsistencies and second, performing molecular dynamics simulation. The final model was compared with well-characterized RNA ligases 2.

Available information on how RNA ligases 2 interact with their substrate and ATP comes from crystal structure analysis and enzymatic assays of trypanosome TbREL1 and bacteriophage T4RNL2. In contrast, the presented ligand-binding mode of DpRNL was inferred from molecular dynamics simulations that were based on an *in-silico* modeled 3D structure of the protein. Homology models built from a template that is very distant in sequence space are usually less reliable and tend to be biased toward the template. Even if the main chains of residues interacting with ATP are correctly placed in the DpRNL model, misplacement of their side chains may influence the simulation of ligand binding. To alleviate these difficulties, we have refined the homology model using extensive MD simulation, and have tested the resulting structure using several metrics (e.g. SAVES, ModFold). The predicted unusual ATP-binding mode in the *Diplonema* protein must be considered with this precautionary note in mind.

### How the postulated rewiring of ATP interactions in DpRNL may have evolved

The present model of DpRNL indicates a reorganization of residue-residue and residue-ATP interactions in the catalytic pocket compared to other ligases, entailing that (i) the ribose is less firmly stabilized than in TbTEL1 and T4RNL2, (ii) the conserved lysine DpRNL_K20 in motif I is pulled away from ATP, and (iii) ATP is now contacted by the conserved lysine DpRNL_K175 in motif V. Such a reshaping would most likely impact steps 1 and 2 of the catalysis (Additional file 1: Figure S1; Additional file 1: Figure S10, see legend for detailed description of the hypothesis).

Evolution of such reorganization in the catalytic pocket of DpRNL would require at least two consecutive steps. We speculate that initially, the nearly neutral mutation of a valine to tyrosine DpRNL_Y161 (at the position corresponding to residue 207 in T4RNL2) was made possible by the subsidiary presence of the lysine DpRNL_K175, which incidentally replaced the original catalytic lysine (DpRNL_K20). In this intermediary step, the system could have reverted back to its previous organization. Yet, the accumulation of mutations in a second step (DpRNL_V177, DpRNL_R173 by genetic drift) led to a state with no way back, in the manner of a ratchet [39]. Such a two-step scenario is archetypal of the constructive neutral evolutionary process [40].

As mentioned before, two residues highly conserved at the structural level are predicted to have a different function in DpRNL compared to orthodox RNA ligases 2. These are the ubiquitous lysine (TbREL_K87|T4RNL2_K35) and arginine (TbREL_R111|T4RNL2_R55), which correspond in the structure alignment to DpRNL_K20 and DpRNL_R41, respectively (Fig. 6). Conservation of the residues in *Diplonema* but not their predicted function raises the question about the underlying selection pressure. Interestingly, the catalytic lysine of proven RNA ligases 2 (e.g., TbREL_K87), has been suggested to also interact with the RNA substrate, notably in the reaction step 3 [41] (Additional file 1: Figure S1). Therefore, we speculate that both DpRNL_K20 and DpRNL_R41, may be subject to a negative selection in favor of conserving a second yet unrecognized role. The key message is that the observation of constant sites across an otherwise diverse family is not necessarily indicative of an identical molecular function of the corresponding residues, as residues can play multiple (structural and catalytic) roles in the corresponding protein [42].

### The biological process involving DpRNL

We found that sequence- and structure-wise, mitochondrial RNA ligases 2 of kinetoplastids are not the closest homologs of DpRNL. Specifically, the 3D-structure model of DpRNL does not fit better the structure of TbREL compared to that of RNA ligases 2 from a bacteriophage or an archaean. Further, phylogenetic analysis of RNA ligases 2 did not group together the kinetoplastid and diplonemid proteins, but placed DpRNL without support next to a member of the heteroloboseans, a group that emerged prior to Euglenozoa. The large distance between kinetoplastid RNA ligases and DpRNL is probably due to a divergent, accelerated evolution and hyper-specialization of both the kinetoplastid and *Diplonema* proteins. Therefore, we cannot extrapolate from TbREL the biological process in which DpRNL may be involved.

At present it is unknown whether or not DpRNL acts inside mitochondria. There is no recognizable signal in the inferred protein sequence indicative for import into mitochondria or any other subcellular localisation. After translation, DpRNL may either remain in the cytoplasm or be imported into mitochondria by one of the cryptic signals reported for proteins of several other eukaryotes [43]. If DpRNL indeed ends up in mitochondria, then its interaction partner must be fundamentally different to those of the kinetoplastid TbREL, because of significant structural differences between the two proteins (e.g. characteristics of the C-terminal domain, the pattern of electrostatic surface potential, and the absence of interacting loops). Our in silico analyses have prepared the ground for determining experimentally the location of DpRNL in the cell, the protein and RNA partners with which it may interact, and ultimately, via ‘guilt by association’ , the biological process in which it participates.

## Conclusion

RNA ligase 2 from bacteriophage T4 is widely used as a tool in molecular biology, in particular for massively parallelized RNA sequencing technologies. Enzyme versions have been engineered with higher efficiency and fidelity than the natural protein. Specifically, the truncated version of the RNA ligase from phage T4 produces less concatemer side products and is 10 times more active than the natural enzyme [23]. Further, attempts have been undertaken to abolish concatemer formation of T4 RNA ligase by directed mutation of specific amino acids (substitution of T4RNL2-K227 by glutamine abolishes reversibility of the second step of the reaction) [34]. Comparative analysis with divergent RNA ligases such as DpRNL are bound to reveal unrecognized evolution-born innovations and to pinpoint residues otherwise not expected to be relevant enzymatically. Our *in-silico* analysis suggests that DpRNL activity relies on structure-function innovations not present in the commonly used RNA ligases, which might reveal suitable for future applications in biotechnology.

## Methods

### Identification of RNA ligase 2

We identified RNA ligase 2 in the draft version of the *D. papillatum* nuclear genome obtained from a Mira V3.4.1.1 [44] assembly of 7.5 million 454 reads at a coverage of ~ 10×. The search was performed with PFAM [27] domain PF09414 present in kinetoplastid RNA ligase employing HMMer 3 [45, 46] using the maximum sensitivity option (parameter --max). We found a single significant hit (E-value = 1.3e-06) in the *Diplonema* sequence matching a hypothetical protein (DpRNL). The identification of the domains characteristic for the RNA ligase 2 family was first performed by analysing the alignment of DpRNL with the PF09414 HMM domain in the HMMer result file, then by a multiple alignment of the two ligase paralogs from four Leishmania species (*L. braziliensis*: LbrM.20.5890 and LbrM.01.0620; *L.mexicana*: LmxM.01.0590 and LmxM.20.1730; *L. major* Friedlin: LmjF.20.1730 and LmjF.01.0590; *L. infantum*: LinJ.01.0610 and LinJ.20.1700) and six Trypanosoma species (*T. brucei* TREU927: Tb09.160.2970 and Tb927.1.3030; *T. brucei* Lister strain 427: Tb427.01.3030 and Tb427tmp.160.2970; *T. brucei* gambiense: Tbg972.1.1840 and Tbg972.9.2300; *T. cruzi* CL Brener Esmeraldo-like: Tc00.1047053506363.110 and Tc00.1047053511585.20; *T. cruzi* CL Brener Non-Esmeraldo-like: Tc00.1047053506975.9 and Tc00.1047053510155.20; *T. congolense*: TcIL3000.1.1450 and TcIL3000.9.1420; *T. vivax*: TvY486_0101350 and TvY486_0901490).

The specificity of PF09414 in detecting RNA ligases 2 was evaluated by comparing the score of all PFAM domains of DNA and RNA ligases against (i) the *Diplonema* candidate RNA ligase, (ii) the two well characterized RNA ligases from *Trypanosoma brucei* TREU927 (TbREL1, Gene ID = Tb927.9.4360 and TbREL2, Gene ID = Tb927.1.3030) downloaded from TriTrypDB [47], and (iii) the RNA ligase from *Naegleria gruberi* (XP_002674912.1), a protist diverging basally to Euglenozoa.

### Three-dimensional structure modeling

The three-dimensional model of DpRNL has been determined by I-Tasser (the Iterative Threading Assembly Refinement program) web server (http://zhanglab.ccmb.med.umich.edu/I-TASSER/) [48] using default parameters (no restraints, no guide or exclusion template). I-Tasser selected the structure of the DNA ligase of Aquifex aeolicus (PDB ID = 3QWU) as the closest structural homolog of DpRNL and proposed five candidate models. Then, we refined the models with ModRefiner [49], and evaluated the quality of the models with tools available from the SAVeS Web server (Structural Analysis and Verification Server http://services.mbi.ucla.edu/SAVES/) and ModFold [30]. From the five models proposed by I-Tasser, we selected the one having the lowest structural variations compared to the template, and the best structural qualities according to SAVeS.

### System preparation for molecular dynamics simulations

Two different molecular dynamics simulation protocol were used for DpRNL. To investigate the stability of our model, we used the apo form of the protein (apo-DpRNL). To examine the interactions between the ligand and the protein, we used DpRNL with bound ATP and Mg^2+^ (DpRNL_ATP+Mg^2+^). In this experiment, we superimposed DpRNL onto TbREL1, the Trypanosoma homolog of DpRNL crystallised with ATP (PDB ID = 1XDN), and manually copied the ATP and Mg^2+^ residues from 1XDN to the corresponding position in DpRNL. We added hydrogens when needed with WHATIF [50] and rendered the file CHARMM compatible by employing the PDB Reader of CHARMM-GUI [51].

### Molecular dynamics simulations

All molecular dynamics (MD) simulations were performed with the Gromacs 4.0.5, 4.6.5, 5.0.1 and 5.0.2 software [52] and CHARMM27 force field [53]. We modified the charmm27.ff force field [54] files in Gromacs to add topology and parameter information for ATP from toppar_all36_na_nad_ppi.str by following the procedure specified in the Gromacs manual (http://www.gromacs.org/Documentation/How-tos/Adding_a_Residue_to_a_Force_Field). Proteins and ligands were solvated in a cubic box of TIP3P water molecules at a distance of 3 nm (30 Å) from the solute. The net charge of the system was neutralized by addition of six chloride ions for the DpRNL apo system, four chloride ions for DpRNL+ATP+Mg^2+^ and five sodium ions for TbKREL1+ATP+Mg^2+^. The cut-off for short-range van der Waals and electrostatic interactions was 1.0 nm (default values), and PME (Particle Mesh Ewald) was used for long-range interactions in all simulations. First, we performed an energy minimisation by steepest descent to remove possible spurious contacts until convergence to a maximum force of 1000 kJ/mol/nm on any atom of the system (850 steps). For all MD simulations, the leap-frog formula was used to integrate the equations of motion. Then two MD equilibrations of 100 ps each (25,000 steps with 2 fs timesteps) were performed with restrained positions of protein and ligand. For the first NVT (constant number of particles, volume, and temperature) equilibration, the temperature was set to 300K using the V-rescale thermostat [55] with separate baths for protein and non-protein atoms. Then, for the subsequent NPT (constant number of particles, pressure, and temperature) equilibration, the Parrinello-Rahman barostat [56, 57] was used in addition to the V-rescale thermostat in order to couple the pressure to 1 bar. Following these pre-production steps, MD simulation productions were performed on apo-DpRNL and on holo-DpRNL loaded with ATP and Mg^2+^.

For DpRNL apo, we performed a 50 ns simulation with 2 fs timesteps. The 2D structure conservation during the simulation period was measured using the timeline plugin of VMD [58]. For DpRNL loaded with ATP and Mg^2+^, we performed MD simulations of 50 and 45 ns in total. During a preliminary simulation, ATP escaped from the catalytic pocket. Therefore, as a precaution, we restrained its position during the initial 15 ns of the production simulation (referred to as ATP-restrained phase), to let the protein equilibrate around the ligand, and after lifting the restriction, the simulation was continued. First, we used the same 15 ns ATP-restrained simulation (15R0) that we extended by four independent 35-ns MD simulations (replicates 15R0 + 35_1 to 15R0 + 35_4). Second, we ran three independent 15-ns restrained simulations (15RI, 15RII and 15RIII) followed by 30 ns MD simulations. When measuring the distance between the two molecules during the initial time interval, we noted that the restraint was used in less than 1 % of the frames for all the predominant replicates (15R0, 15RI, 15RII) and in 18 % of the frames for the deviant replicate (15RIII) (Additional file 1: Figure S8). As an anchor of the restraint, we chose DpRNL_F101 because first, this residue is highly conserved among ligases; second, it is positioned deeply inside the catalytic pocket; and third, in *Trypanosoma* TbREL1, the adenine of ATP has been shown to make π-stacking interactions with the homologous position, TbREL1-F209 [25]. We set a distance restraint of 0.3 nm around the initial distance ri between each pair of atoms from the phenyl group of DpRNL_F101 and the pyrimidine ring of the adenine, meaning that there is a component for the restraint added to the potential energy function for *r*_i_ >*r*_i_ + 0.30 nm and *r*_i_ <*r*_i_ - 0.30 nm. Three distances are set: *r*_0_ = *r*_i_ - 0.30 nm, *r*_1_ = *r*_i_ + 0.30 nm and *r*_*2*_ = *r*_*1*_ + 1 nm. The potential for the distance restraints is quadratic below *r*_0_ and between *r*_1_ and *r*_2_, and linear above *r*_2_.

To test whether inserting ATP + Mg^2+^ in the catalytic pocket of DpRNL leads to a realistic positioning of the ligands, we performed a control experiment on TbREL1. To prepare the system, we replaced the selenomethionine used for crystallisation with methionine, then we ran an MD simulation first on the apo protein for 15 ns. Then, we used the structure from the last frame of the previous simulation as a starting point, inserted ATP + Mg^2+^ into the molecule, and ran a simulation for 30 ns.

### Exceptional residues

In order to identify exceptional residues in the candidate RNA ligase of *Diplonema*, we computed a score measuring how unexpected each residue of the protein is. Using the I-Tasser model of DpRNL as the query structure, we searched for structural “neighbors” with DALI (http://ekhidna.biocenter.helsinki.fi/dali_server/, [59]): we selected 23 unique RNA and DNA ligases whose structure have the highest percentage of identity and the lowest Root Mean Square Deviation (RMSD), and performed a multiple structural alignment including DpRNL. For subsequent computations, we used the alignment without expanding the gaps, meaning that inserted segments relative to DpRNL are hidden. For each position in the 23 proteins, we computed the entropy s as given by [60] which represents the diversity of amino acids for a given position. The entropy *s* at position *l* is *s*(*l*) = − Σ_*i*=1_^6^*P*_*i*_(*l*) logP_i_(l) where i is the category of amino acid (1: aliphatic, {AVLIMC} 2: aromatic {FWYH}, 3: polar {STNQ}, 4: positive {KR}, 5: negative {DE}, 6: special {GP}), and *P*_*i*_(*l*) is the proportion of amino acids belonging to category i at position l. At a given position, amino-acid categories for which *P*_*i*_(*l*) is null are ignored. If the entropy is low, then the position is conserved among family members. The entropy is set arbitrarily to 0 when the position in the multiple alignment contains more than 50 % gaps. We designed an exceptionality score S at position l for amino acids of DpRNL as Sl = (*P*_max_(*l*) - *P*_*i*_(*l*)) / *s*(*l*) where *P*_*i*_(*l*) is the proportion in the previously computed multiple alignment of the amino acid observed at position l for DpRNL, and P_max_(l) is the proportion of the most abundant amino-acid category (the category that we expect).

### 3D model analyses

Trajectory analyses were performed with R [61], VMD [62] and PyMOL [63]. Hydrogen bonds were computed using VMD with a distance cutoff of 3.0 Å and an angle cutoff of 30°. The evolution of the secondary structure [58] was computed via the timeline plugin of VMD based on the STRIDE algorithm [64]. The conservation surface was colored with the web server ConSurf [65] using the structural multiple alignment performed by DALI as input and with the Bayesian method for computing the evolutionary rate [66].

The electrostatic potential of the molecule was computed by the classical calculation using the last frame of the simulation, employing the APBS web server (http://www.poissonboltzmann.org/) [67–69] and visualized using the dedicated APBS plugin of PyMOL. The isovalue cut-off for the analyses was set to +5*k*_B_*T*/*e* (blue) and +5*k*_B_*T*/*e* (red). For DpRNL, this procedure was sufficient to reveal a large region with positive potential, having the propensity to bind RNA. In contrast, for TbREL1, the classical potential calculation (using Delphi [25]) identified only small positive patches. To find a positive region sufficiently large for RNA binding in TbREL1, the authors had to calculate an ensemble average on their 70-ns simulation [31].

### Expression

The expression of the gene coding for DpRNL was assessed by mapping RNA-seq reads from a total-RNA library of *D. papillatum* onto the contig carrying the gene. Library construction and read processing have been described earlier [19]. Cutadapt version 1.2.1 [70] was used to remove adapters at 5′ and 3′ termini of reads with an error rate of 0.1 and to clip low-quality sequences with a threshold of 20. Reads <20 nt were discarded, leaving 29 million paired reads, which were mapped with Bowtie2 [71] onto the 1314-nt long contig containing the DpRNL reading frame. Output files in sam format were subsequently transformed into ‘bam’ files with SAMtools version 1.4 [72]. Alignments were visualized with tablet version 1.13.05.17 [73].

### Phylogenetic reconstruction of RNA ligases 2 from Excavata

We identified RNA ligase 2 proteins in Excavata species by searching with the same PFAM HMM PF09414 as used for *Diplonema*. Sequences were aligned using MAFFT with option "--localpair" (for distantly related species with a single alignable domain). The multiple alignment was refined by successive re-alignment of the sequences on a guiding hmm model built from the alignment with HMMer 3 [45, 46]. The best scoring alignment according to HMMer was selected and filtered with an in-house script to retain positions with less than 30 % gaps and a conservation score greater than 8 as given in the stockholm format. We reconstructed the phylogeny with RaXMLHPC v.7.2.6, a maximum likelihood method, using a gamma distribution to model the heterogeneity of substitution rate over sites and the WAG substitution matrix. A Bootstrap analysis of 100 runs was performed to assess the significance of each node.

## Availability of supporting data

The sequence of DpRNL is available under Genbank accession number KT828338. The 3D model is included as additional files in PDB format. Alignment is available on request.

## Additional files

Additional file 1:
**Supplementary data.** Supplementary result, figures and tables for DpRNL identification, phylogenetic study, 3D model properties, and complementary analyses of MD simulations (DOC 3297 kb) Additional file 2:
**DpRNL model.** Atomic coordinates of DpRNL model. (PDB 258 kb) Additional file 3:
**ITasser threading of DpRNL on 3QWU.** Atomic coordinates of DpRNL and 3QWU model. (PDB 11 kb)

## Abbreviations

2D: Secondary structure
3D: Tertiary structure
ATP: Adenine triphosphate
DpRNL: RNA ligase 2 from *Diplonema papillatum*
HMM: Hidden Markov model
MD: Molecular dynamics
PFAM: Protein FAMily database
T4RNL2: RNA ligase 2 from bacteriophage T4
TbREL1: RNA ligase 2 from *Trypanosoma brucei*, paralog of KREL1, Tb927.9.4360
