# Supplementary material for: Three-dimensional structure model and predicted ATP interaction rewiring of a deviant RNA ligase 2
Source: BMC Struct Biol. 2015 Oct 9;15:20. doi: 10.1186/s12900-015-0046-0 (PMC4599027; doi:10.1186/s12900-015-0046-0)
Supplement: Additional file 1: — Supplementary data. Supplementary result, figures and tables for DpRNL identification, phylogenetic study, 3D model properties, and complementary analyses of MD simulations (DOC 3297 kb) [file 12900_2015_46_MOESM1_ESM.doc]

ADDITIONAL FILE 1: SUPPLEMENTARY DATA

**Figure S1**. Catalytic steps of the ligation reaction performed by an RNA ligase 2

**Figure S2**. Alignment of the PF09414-Rnl2 HMM model with DpRNL

Figure S3. RMSD (Root Mean Square Deviation)

Figure S4. Define Secondary Structure of Proteins (DSSP) calculated for DpRNL with the tool VMD.

**Figure S5**. Hydrophobicity of residues on *Pyrococcus abyssi* and *Diplonema* 3D representation

**Figure S6.** Phylogenetic tree of RNA ligases 2 from Excavata

Figure S7. RMSF (Root Mean Square Fluctuation) of DpRNL apo backbone, 50 ns simulation

Figure S8. Number of violations of distance restraints during the 15-ns of the four ATP-restrained simulation replicates

Figure S9. Design of MD simulations performed

Figure S10. Proposed model for ATP interactions rewiring in DpRNL

Figure S11. Catalytic pocket for the deviant MD simulation

Table S1. Comparison of DpRNL with close structural homologs of interest

Table S2. Comparison of ATP interaction with DpRNL and TbREL1


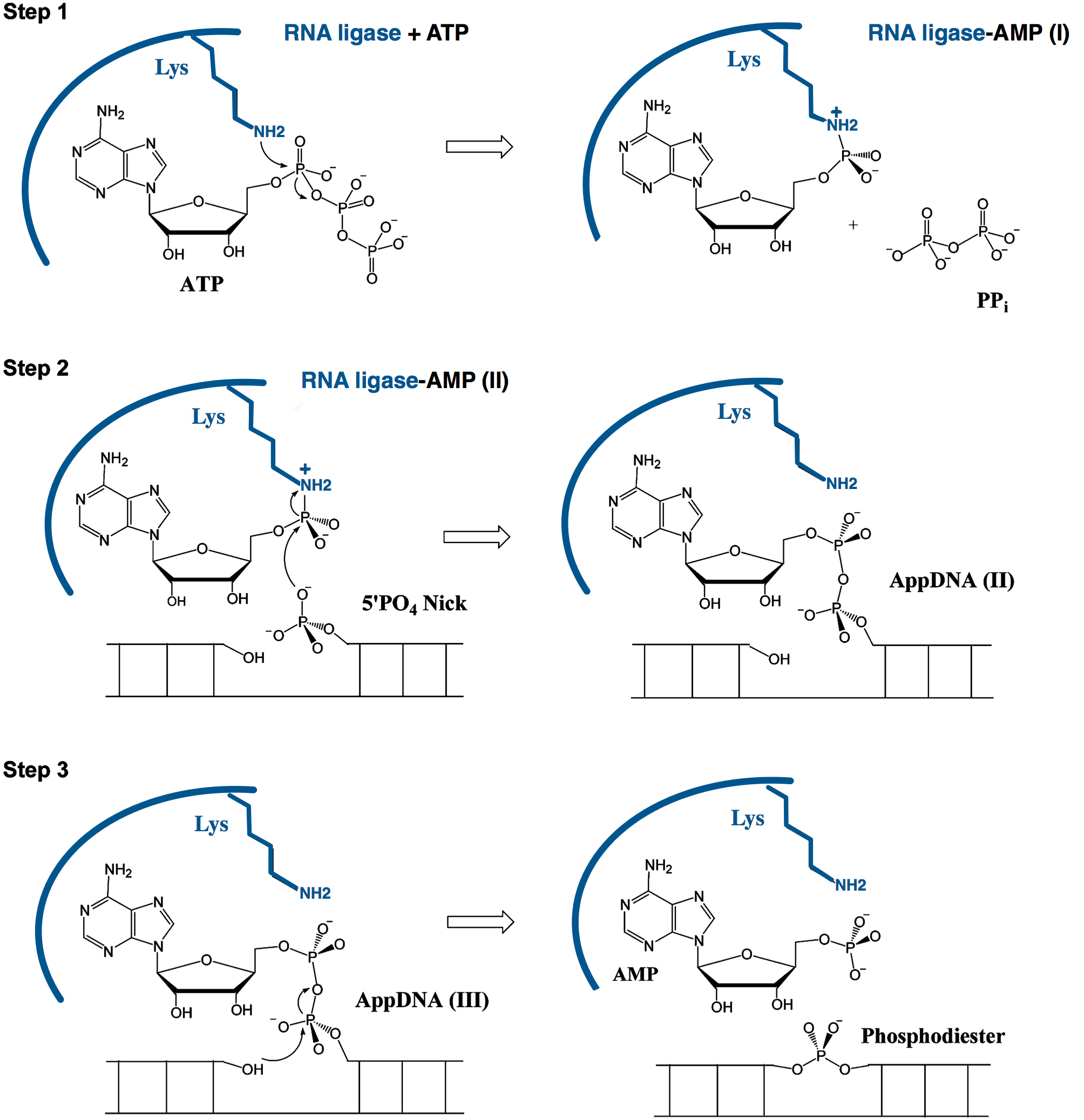


Figure S1. Catalytic steps of the ligation reaction performed by an RNA ligase 2 adapted from [1]


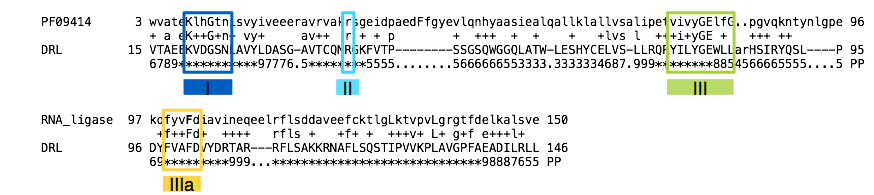


Figure S2. Alignment of the PF09414-Rnl2 HMM model with DpRNL. The alignment was produced by HMMer [2, 3]. Four Nucleotidyltransferase sub-domains are highlighted.

A. B.


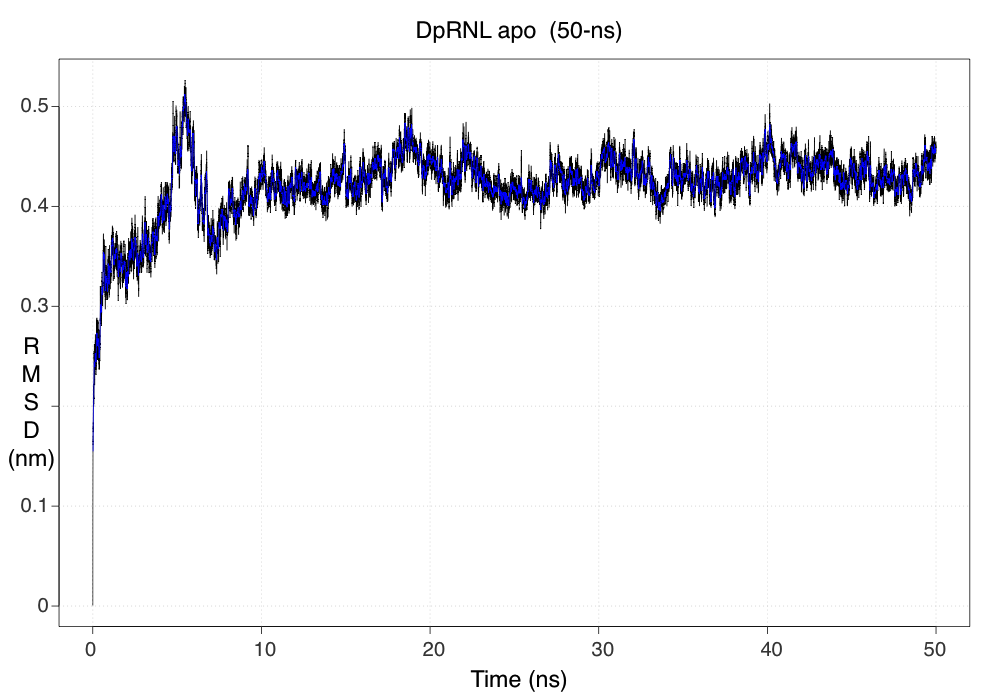

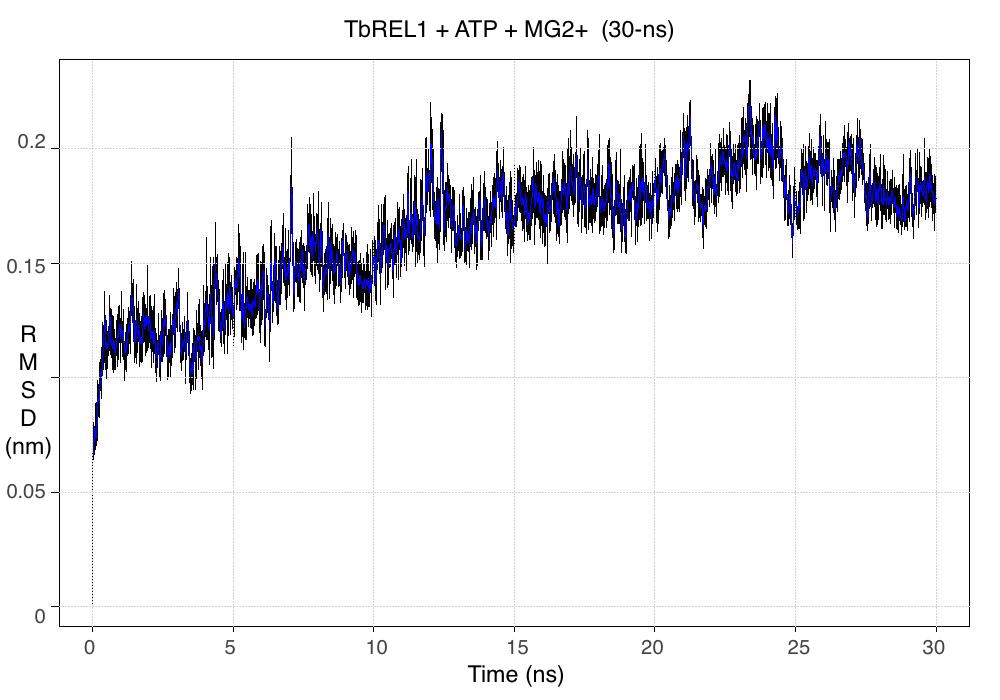


C.


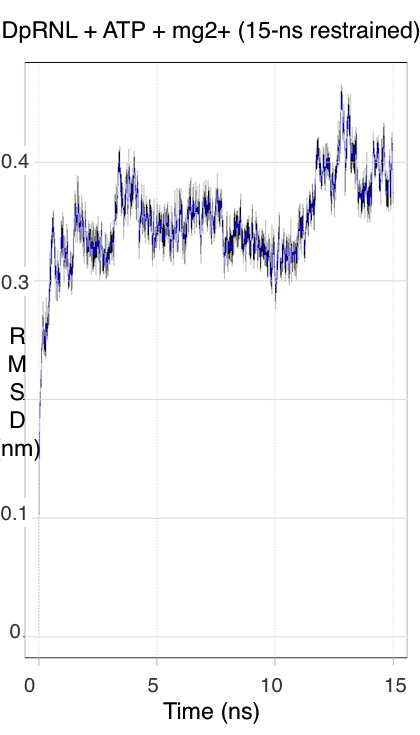

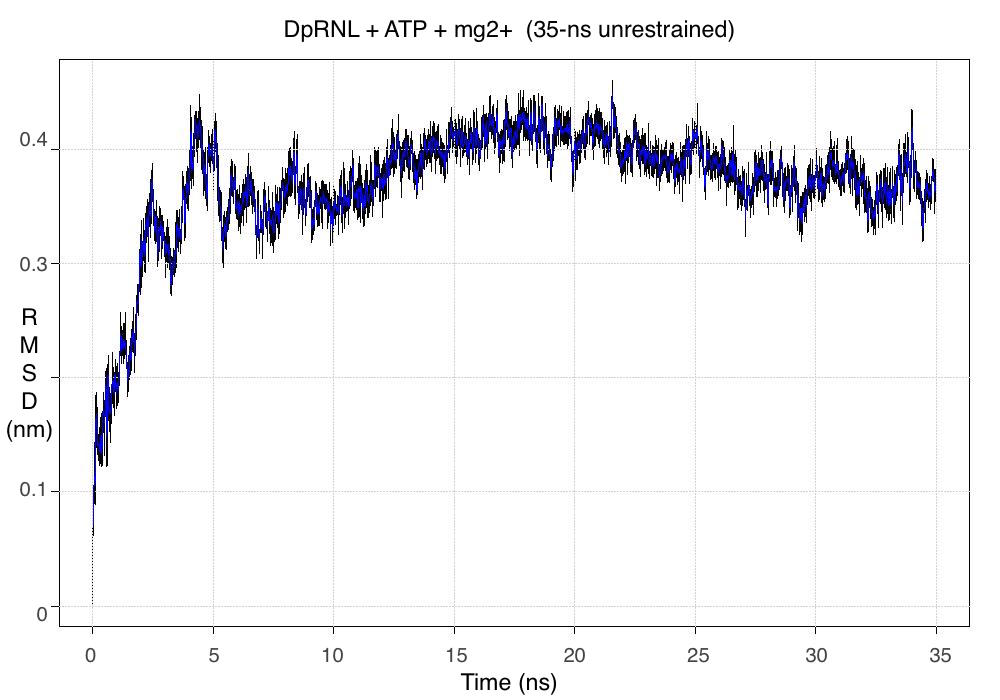


Figure S3. RMSD (Root Mean Square Deviation). RMSD-Backbone after least-square fit to backbone A. DpRNL apo backbone, 50 ns simulation, B. TbREL + ATP + Mg^++^, 30 ns simulation, C. DpRNL + ATP + Mg^++^ , 50-ns (replicate 15R0+35_1) .


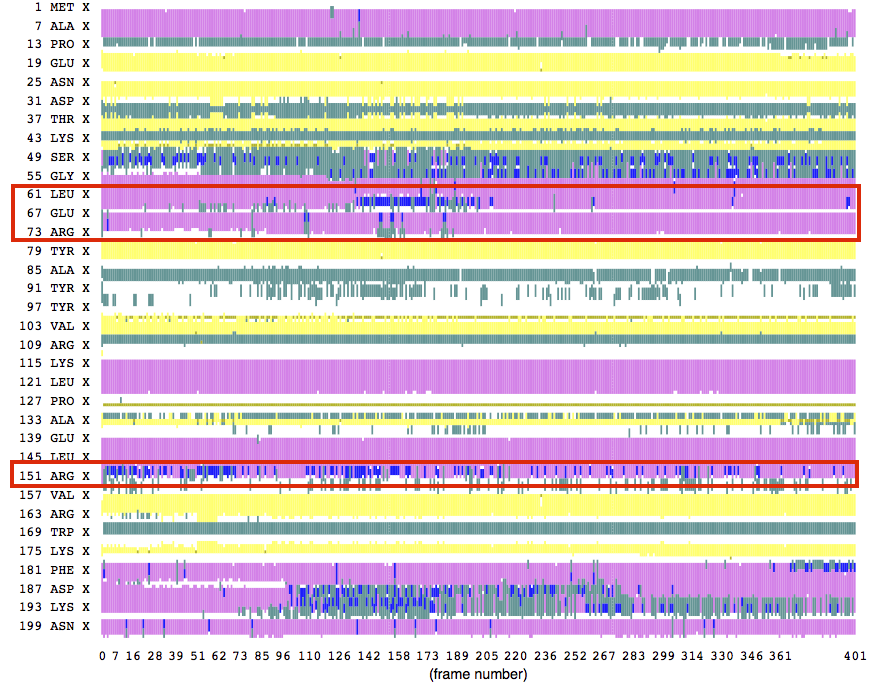


**Figure S4.** Define Secondary Structure of Proteins (DSSP) calculated for DpRNL (replicate 15R0+35_1) with the tool VMD. Each amino acid of the ligase (vertical) is assigned a secondary structure state for every frame of the simulation (horizontal). Pink, α helices; blue, 3-10 helices; yellow, β strands; green, turns; and white, other loops. Flexible helices are highlighted in red.

|  | Cartoon representation | Cartoon and surface |
| --- | --- | --- |
| **2VUG**  *Pyrococcus abyssi* | 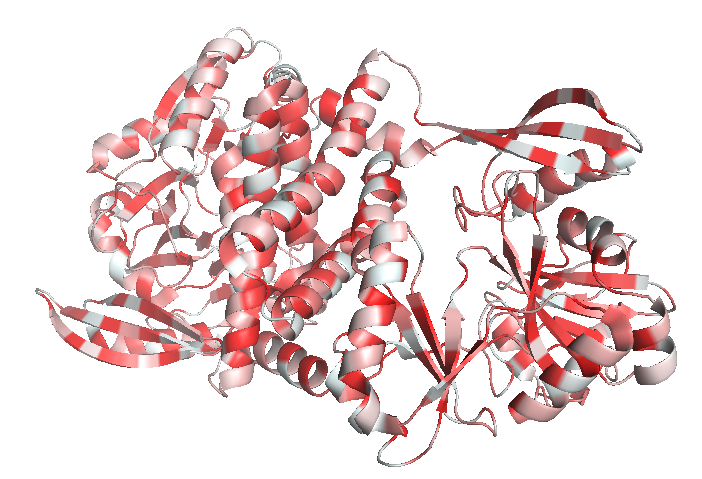 | 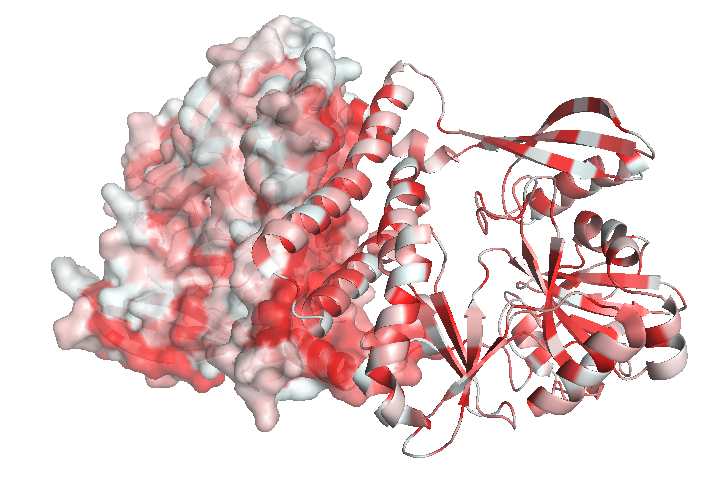 |
| **2VUG - Interaction helices**  *Pyrococcus abyssi*  Monomer with only interaction helices highlighted | 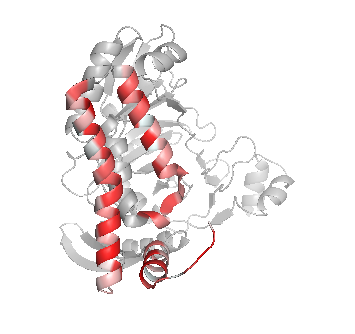 | 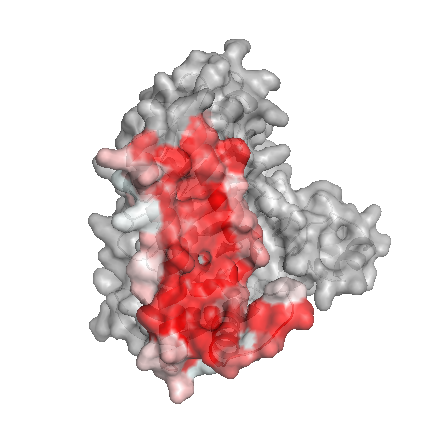 |
| **DpRNL**  *Diplonema papillatum* | 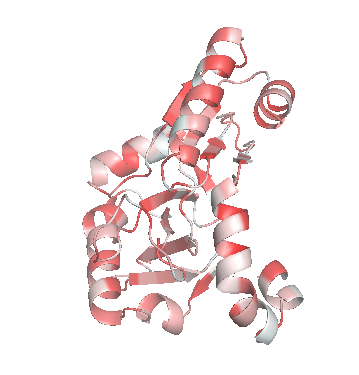 | 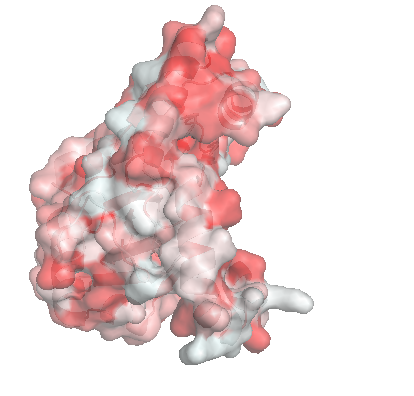 |
| **DpRNL - C-terminal helices**  *Diplonema papillatum* | 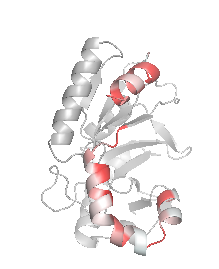 | 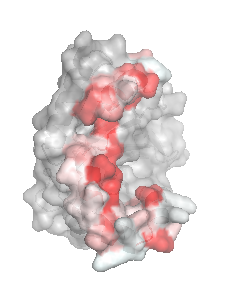 |

Figure S5. Hydrophobicity of residues represented by different shades of red according to the Eisenberg hydrophobicity scale [4] on Pyrococcus abyssi and Diplonema 3D representation. The hydrophobic alpha helices allowing dimerisation are visible in the archaean structure whereas the corresponding region in DpRNL’s model does not display such an hydrophobic surface.


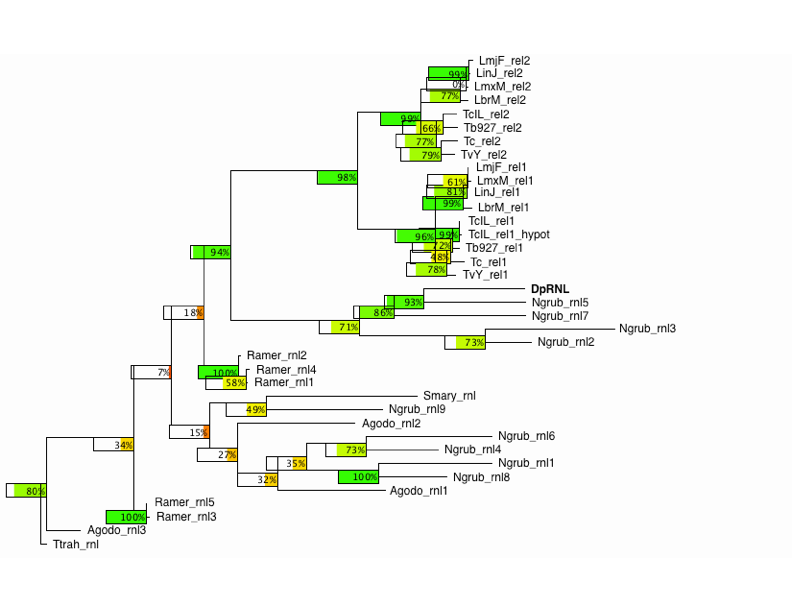


Figure S6. Phylogenetic tree of RNA ligases 2 from Excavata. Leishmania major strain Friedlin (LmjF, rel1: LmjF.01.0590, rel2: LmjF.20.1730), Leishmania infantum strain JPCM5 (LinJ, rel1: LinJ.01.0610, rel2: Linj.20.1700), Leishmania mexicana strain MHOM/GT/2001/U1103 (LmxM, rel1: LmxM.01.0590, rel2: LmxM.20.1730), Leishmania braziliensis strain MHOM/BR/75/M2904 (LbrM, rel1: LbrM.01.0620, rel2: LbrM.20.5890), Trypanosoma congolense strain IL3000 (TcIL, rel1: TcIL3000_9_1420, rel1_hypot: TcIL3000_0_36090, rel2: TcIL3000_1_1450), Trypanosoma brucei strain TREU927 (Tb927, rel1: Tb927.9.4360, rel2: Tb927.1.3030), Trypanosoma cruzi strain CL Brener Non-Esmeraldo-like (Tc, rel1:TcCLB.510155.20, rel2: TcCLB.506975.9), Trypanosoma vivax strain Y486 (TvY, rel1: TvY486_0901490, rel2: TvY486_0101350), Diplonema papillatum (DpRNL), Naegleria gruberi (Ngrub, rnl1: XP_002669886.1, rnl2: XP_002670045.1, rnl3: XP_002672316.1, rnl4: XP_002672691.1, rnl5: XP_002674912.1, rnl6: XP_002676530.1, rnl7: XP_002678251.1, rnl8: XP_002679286.1, rnl9: XP_002679917.1), Reclinomonas americana (Ramer, rnl1: Contig155.33 [1608 - 145], rnl2: Contig383.16 [7789 - 6296], rnl3: Contig411.2 [830 - 2062], rnl4: Contig54.25 [1893 - 3302], rnl5: Contig7385.1 [4474 - 3215]), Sawyeria marylandensis (Smary, rnl: SML00003242), Andalucia godoyi (Agodo, rnl1: comp2286_c0_seq2_1, rnl2: comp3909_c0_seq1_5, rnl3: comp8362_c0_seq3_5), Thecamonas trahens (Ttrah, rnl: AMSG_03775). RNA editing ligases are abbreviated as «rel» and hypothetical RNA ligases as «rnl».


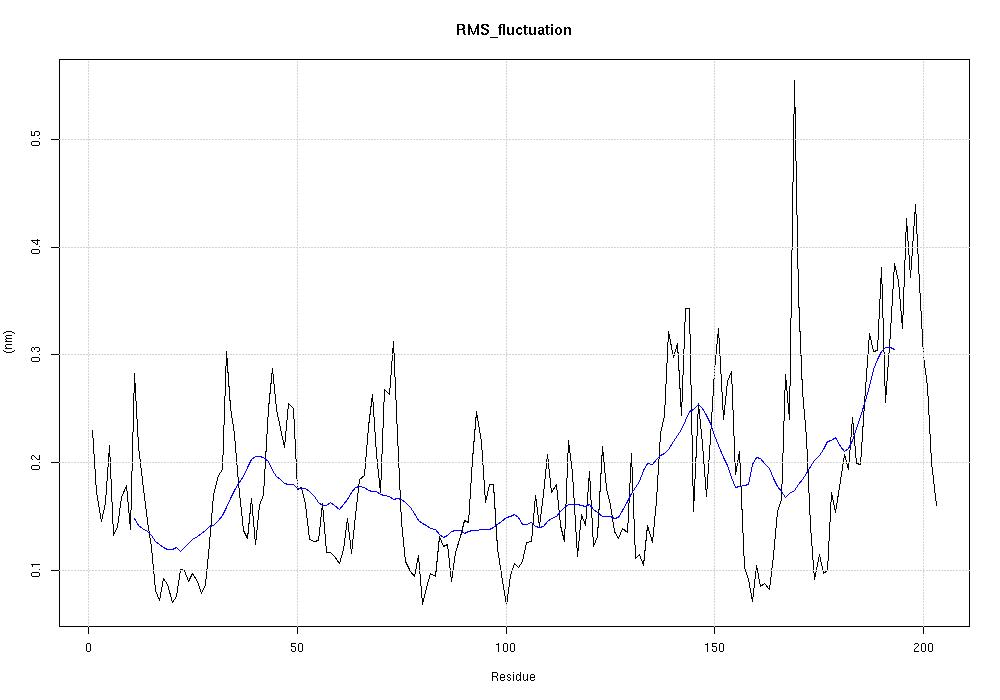


Figure S7. RMSF (Root Mean Square Fluctuation) of DpRNL apo backbone, 50-ns simulation. Blue line: smoothed average.

| 15R0  # frames with violations = 1 | 15RI  # frames with violations = 0 |
| --- | --- |
| 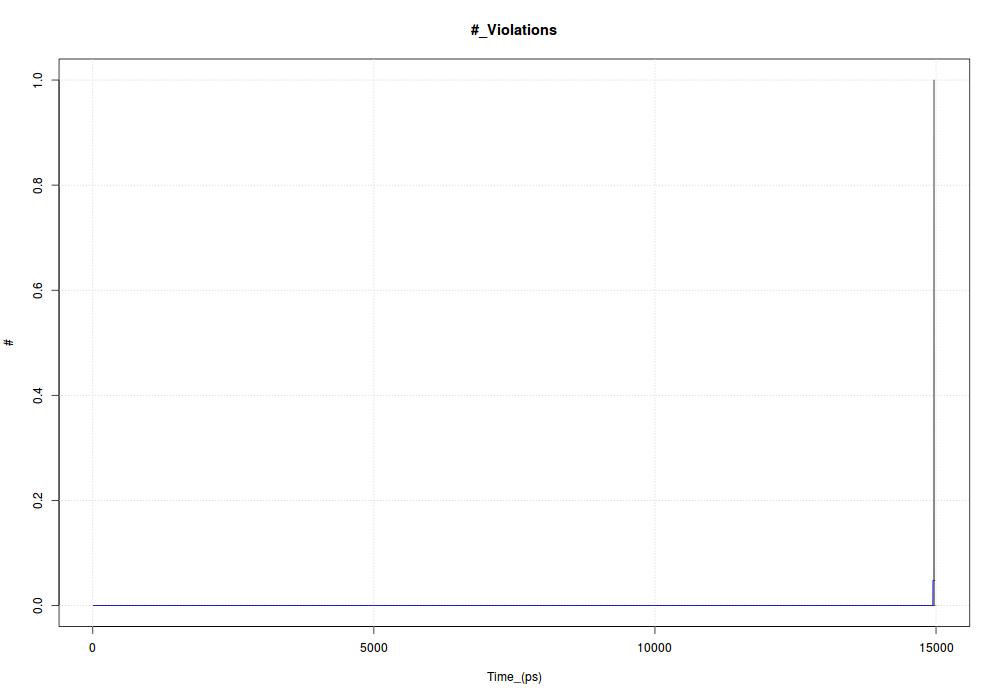 | 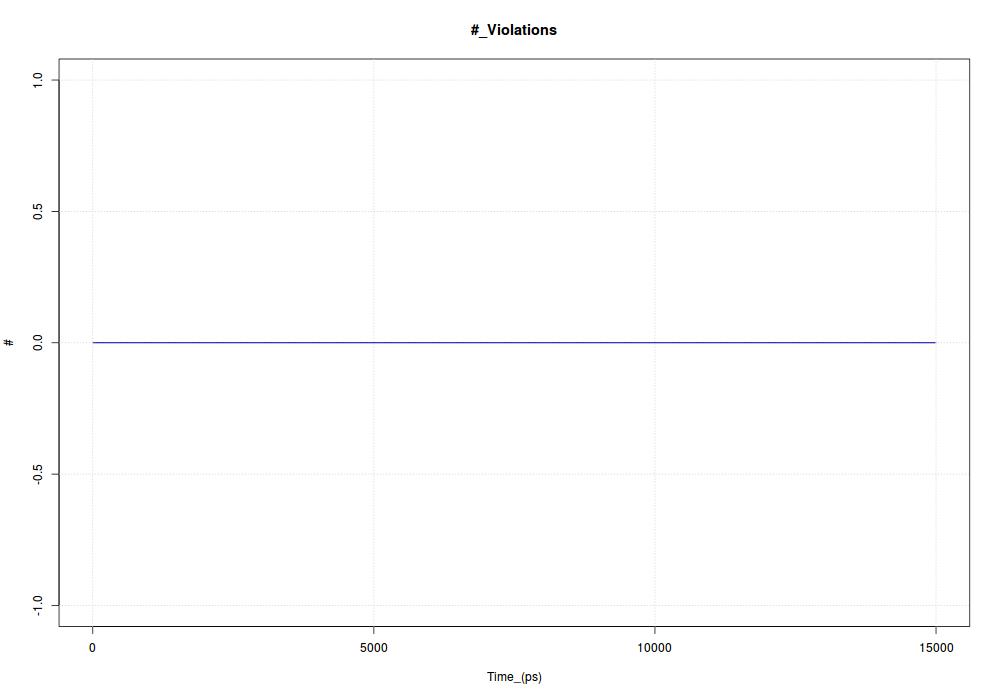 |
| 15RII  # frames with violations = 12 | 15RIII («deviant replicate»)  # frames with violations = 1358 |
| 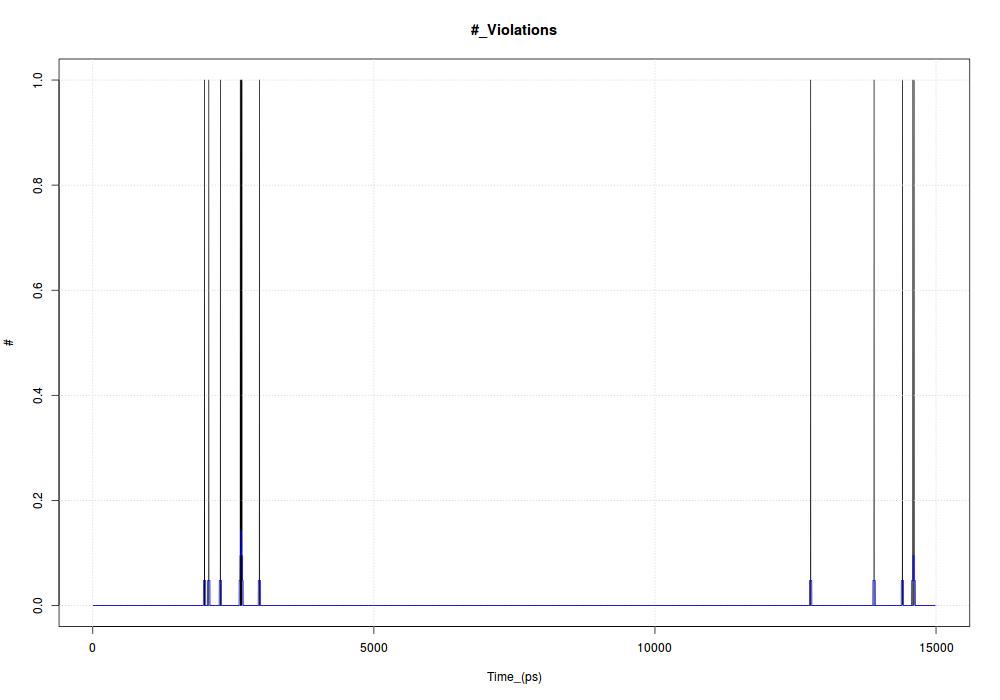 | 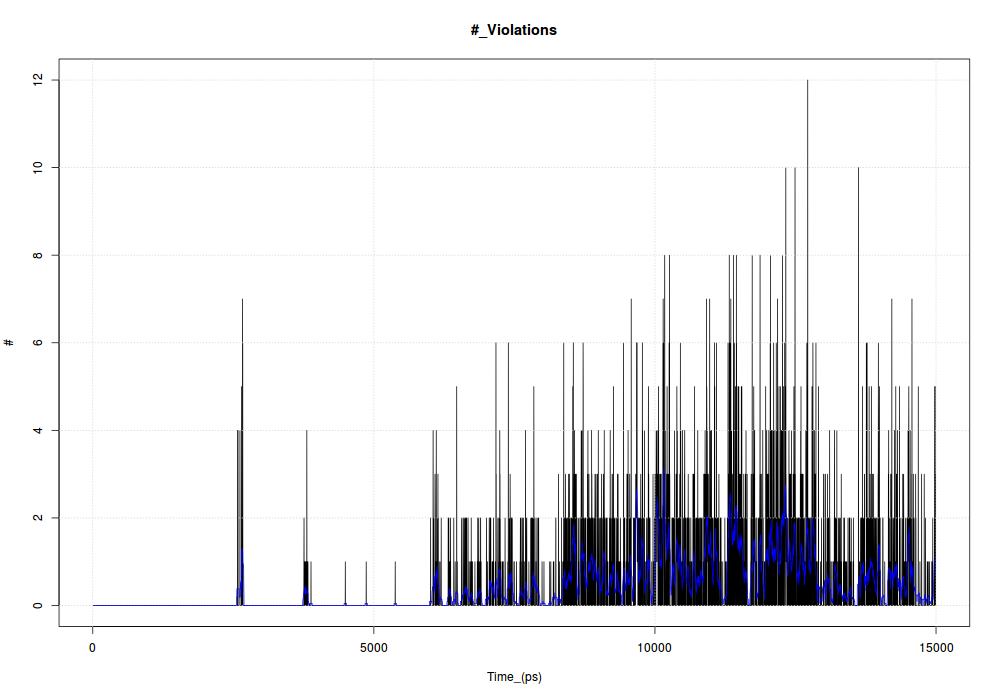 |

Figure S8. Number of violations of distance restraints during the 15-ns of the four ATP-restrained simulation replicates. No force is applied to the system to restrain the distance between the centroid of the phenyl group of DpRNL-F101 and the pyrimidine group of ATP between the green line (r0) and the red line (r1). During this simulation, the restriction did not come into effect.


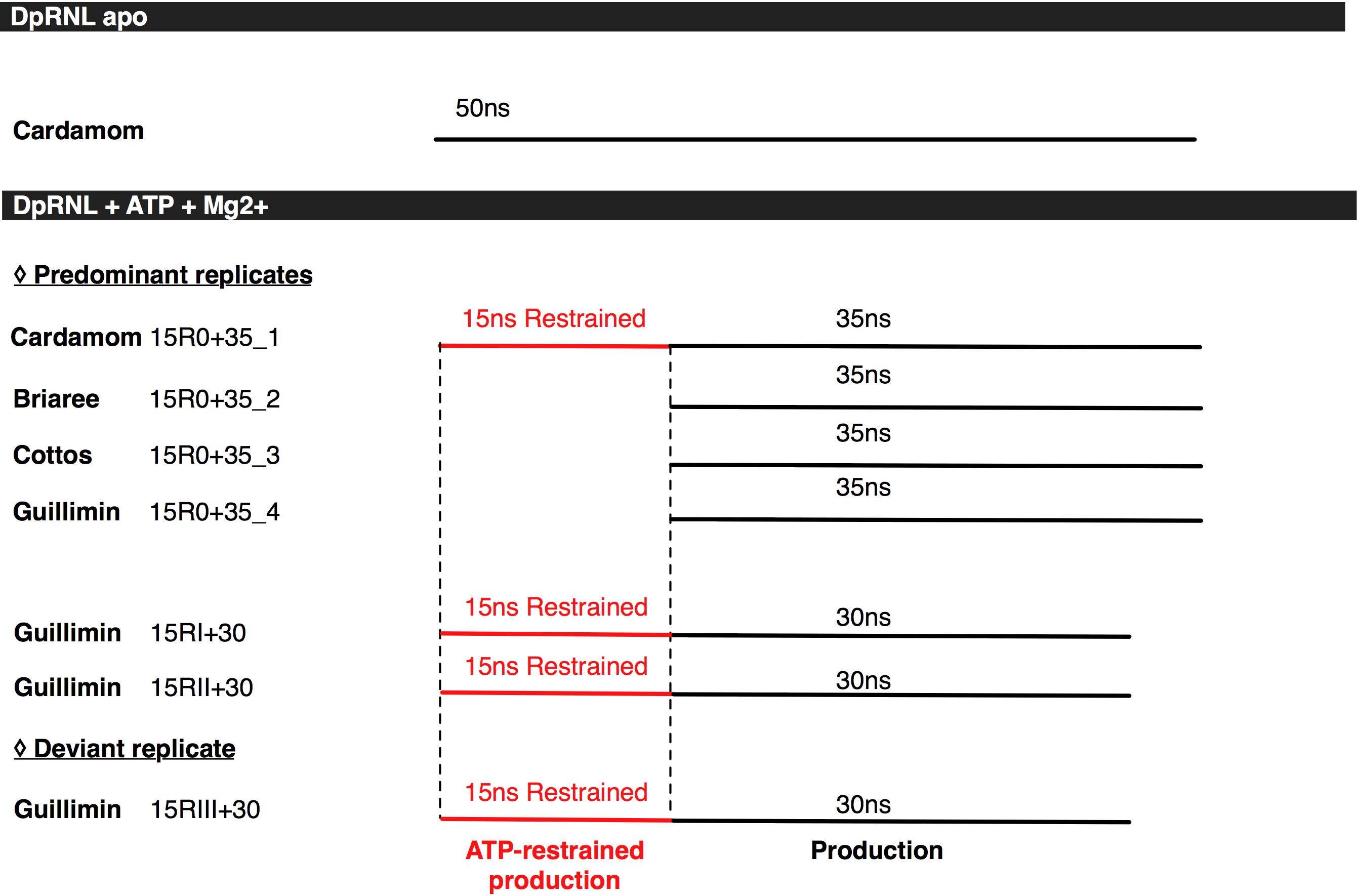


Figure S9. Design of MD simulations performed. A single MD simulation was performed on the apo enzyme. For DpRNL loaded with ATP and Mg2+, four independent 15-ns ATP-restrained simulation (in red, ATP-restrained production phase) were performed. Following the first (15R0-*) ATP-restrained simulation, 4 independent MD simulations (production phase) of 35 ns were run. For the other ATP-restrained replicates (15RI-*, 15RII-*, 15RIII-*) a single production MD simulation of 30 ns was performed. Cardamom, Briaree, Cottos, Guillimin designate the server used which corresponds to different versions of Gromacs: Gromacs 4.0.5 for Cottos, 4.6.5 for Cardamom, 5.0.1 for Briaree and 5.0.2 for Guillimin.


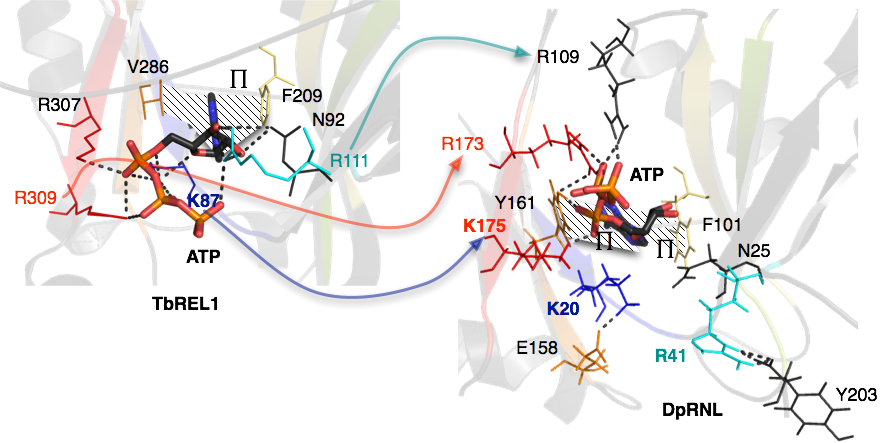


Figure S10. Proposed model for ATP interactions rewiring in DpRNL. Change in the function of a residue is depicted by an arrow. According to our predictions, the most peculiar feature of DpRNL is the reorganization of residue-residue and residue-ATP interactions in the catalytic pocket compared to other ligases. In fact, one of the rare residue in the ATP interaction network of DpRNL that is both structurally and functionally conserved appears to be DpRNL_F101, on which the adenine base is stacked canonically. Reshaping of DpRNL is thought to impact steps 1 and 2 of the catalysis (Supplementary Figure S1). We have shown that in the DpRNL model, the adenine of ATP is sandwiched between two aromatic residues (the highly conserved DpRNL_F101 and the ‘exceptional’ DpRNL_Y161) via double π-stacking, and that the orientation of the base in the catalytic pocket has been shifted as a result. This shift entails several consequences. First, the ribose is less firmly stabilized than in TbTEL1 and T4RNL2, by only two indirect interactions (with DpRNL-N25 and DpRNL-E81). Second, the conserved lysine DpRNL_K20 in motif I is contacted by H-bonds and salt bridges and pulled away from ATP, so that now, ATP could be contacted by the conserved lysine DpRNL_K175 in motif V which is not contacted by any other noncovalent interaction. This latter residue is therefore proposed to attack the α phosphorus of ATP and establish the covalent bound with AMP in reaction step 1 (blue arrow). Third, the triphosphate tail of ATP is stabilized by the exceptional residues DpRNL_R173 rather than by the amino acid in DpRNL position 177, which is usually basic (TbTEL1_R_309|T4RNL2_K227, red arrow) but a valine in the Diplonema protein. Finally, the conserved arginine DpRNL_R41, instead of forming H-bonds with the sugar and the polyphosphate tail of ATP as do the structural counterparts of this residue in other RNA ligases 2 (TbREL1-R111|T4RNL2_55, cyan arrow), interacts with the most C-terminal residue DpRNL_Y203, thus restraining the motion of the C-terminal domain. Note that in the structure of the full-length T4RNL2, interactions between the N-terminal and C-terminal domains have not been observed [1].


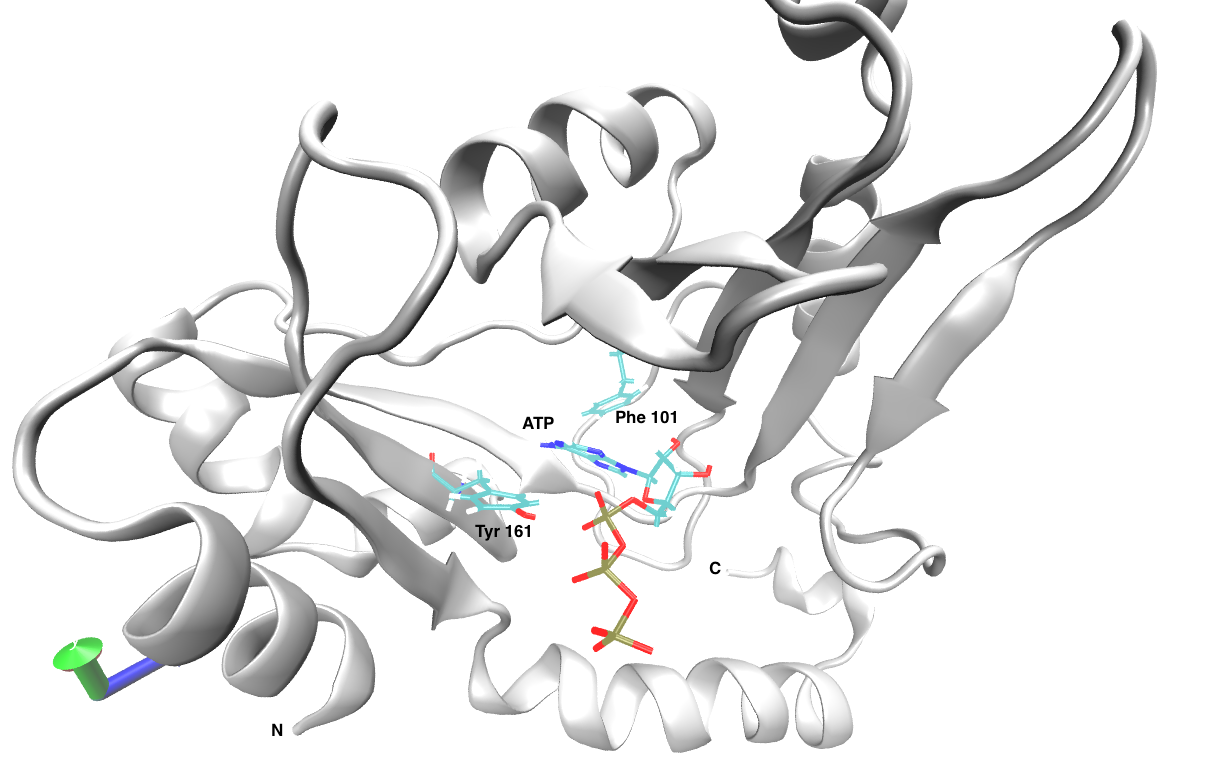


Figure S11. Catalytic pocket for the deviant MD simulation

| **RNA/DNA ligases** |  | **ITasser threading template (LOMETS)** | | | **ITasser**  **(TM-align)** | **Refined DpRNL** | | | |
| --- | --- | --- | --- | --- | --- | --- | --- | --- | --- |
|  | **Length** | **Ident.**  **%** | **Cov.**  **%** | **Z-score** | **TM-score** | **Aligned length** | **RMSD** | **Ident.**  **%** | **TM-score**  **norm. to DpRNL** |
| **3QWU - *Aquifex aeolicus* putative DNA ligase** | 366 | 19 | 96 | 3.13 | 0.878 | 189 | 2.19 | 19.0 | **0.842** |
| 2VUG - *Pyrococcus abyssi* RNA ligase homodimeric | 373 | -/- | -/- | -/- | 0.854 | 187 | 2.40 | 21.4 | 0.823 |
| 1S68 - *Enterobacteriophage T4* 2-233 + AMP | 233 | 21 | 90 | 1.72 | 0.751 | 175 | 3.21 | 18.9 | 0.681 |
| **1XDN - *Trypanosoma brucei* RNA ligase** | 262 | 24 | 89 | **3.52** | 0.745 | 176 | 3.38 | 19.3 | 0.678 |

Table S1. Comparison of DpRNL with close structural homologs of interest from the output of ITasser and by structural comparison of the refined model of DpRNL. When many threading templates for the same template were provided by ITasser, we selected the one with the highest Z-score. Ident. : percentage of identity in the aligned region, Cov: Coverage.

|  | **Motif** | **Protein TbREL1** | **Mean (Std Dev)** | **Protein DpRNL** | **Mean (Std Dev)**  **For all replicates** | **Mean (Std Dev)**  **For deviant MD** |
| --- | --- | --- | --- | --- | --- | --- |
| ATP:PA | I | K87:NZ | 4.04 (0.09) | K20:NZ | **7.7 (0.5)** | 4.4 (0.8) |
| ATP:Adenine | IIIa | F209 | 3.87 (0.23) | F101 | **4.1 (0.01)** | 5.8 (0.3) |
| ATP:Adenine | IV |  | - | Y161 | **4.1 (0.00)** | 5.5 (0.4) |
| ATP:PA | V | K307:NZ | - | K175:NZ | **4.3 (0.01)** | 3.8 (0.2) |

Table S2. Comparison of ATP interaction with DpRNL and TbREL1. Mean distances between residues are reported in angstrom for the six replicates and the deviant replicate.

REFERENCES

1. Nandakumar J, Shuman S, Lima CD: **RNA ligase structures reveal the basis for RNA specificity and conformational changes that drive ligation forward**. *Cell* 2006, **127**:71–84.

2. Finn RD, Clements J, Eddy SR: **HMMER web server: interactive sequence similarity searching.** *Nucl Acids Res* 2011, **39**(Web Server issue):W29–37.

3. Eddy SR: **Accelerated Profile HMM Searches**. *PLoS Comput Biol* 2011, **7**:e1002195.

4. Eisenberg D, Schwarz E, Komarony M, Wall R: **Amino acid scale: Normalized consensus hydrophobicity scale**. *J Mol Biol* 1984, **179**:125–142.
